# Clinical benefits and challenges of ecological momentary assessment in individuals who self-injure and seek mental health treatment

**DOI:** 10.1016/j.ijchp.2025.100618

**Published:** 2025-09-23

**Authors:** Rafaël A. Bonnier, Joanne R. Beames, Laurence Claes, Olivia J. Kirtley, Lena de Thurah, Jeroen D.M. Weermeijer, Lotte Uyttebroek, Mirthe Luijsmans, Inez Myin-Germeys, Glenn Kiekens

**Affiliations:** a Center for Contextual Psychiatry, KU Leuven, Belgium; b Faculty of Psychology and Educational Sciences, KU Leuven, Belgium; c Faculty of Medicine and Health Sciences, University of Antwerp, Belgium; d Department of Medical and Clinical Psychology, Tilburg University, the Netherlands

**Keywords:** Ecological momentary assessment, Experience sampling method, Non-suicidal self-injury, Mental health treatment, Clinical psychology

## Abstract

**Introduction:**

Non-suicidal Self-Injury (NSSI) is a prevalent transdiagnostic behavior. The use of Ecological Momentary Assessment (EMA) shows clinical potential, but the potential utility for individuals who self-injure remains unclear. This prospective study evaluates self-reported benefits (e.g., self-insight and self-efficacy) and challenges (e.g., beep disturbances and emotional discomfort) associated with using EMA among treatment-seeking individuals with past-month NSSI.

**Methods:**

In this cohort study, 124 treatment-seeking adolescents and adults who self-injure completed a 28-day EMA protocol with six daily assessments of emotions, cognitions, and behaviors (including self-injury). After one month, participants completed an EMA feedback survey.

**Results:**

A total of 98 patients completed the feedback survey (Response Rate = 79.03%). Average EMA compliance was 74.87% (SD = 18.78) and decreased linearly across time. Four in five patients (78.57%) reported experiencing at least one benefit. After using EMA, 32.65% reported increased general self-insight, 64.58% reported increased NSSI-specific self-insight, 9.28% reported increased general self-efficacy, and 41.67% reported improved self-efficacy to resist NSSI. Across the sample, 7.29% experienced EMA in treatment as tiring, stressful, at times overwhelming, and not enjoyable. Higher levels of emotional discomfort were significantly associated with lower compliance (*r*=-0.29, *p*=.004), higher beep disturbance (*r*=.37, *p* < .001), and lower general self-insight (*r*=-0.28, *p*=.006). When participants felt more overwhelmed by their emotions than usual, they also reported higher beep disturbance within the same and the next assessment.

**Conclusion:**

Although the use of EMA in treatment may evoke emotional discomfort in patients, it may help promote NSSI-specific self-insight and self-efficacy outside the therapy room in patients who self-injure.

## Introduction

Non-suicidal self-injury (NSSI), defined as the deliberate destruction of one’s own body tissue without suicidal intent (e.g., cutting or burning oneself; International Society for the Study of Self-Injury, 2024), is a pervasive mental health challenge among both adolescents and adults worldwide. Approximately 17-23% of adolescents and young adults (Gillies et al., 2018; Kiekens et al., 2023b) and 3-6% of adults (Liu, 2023; Plener et al., 2016; Swannell et al., 2014) report a history of NSSI. The presence of NSSI is uniquely associated with an increased risk for psychiatric disorders (Kiekens et al., 2023b; Wilkinson et al., 2018), suicidal thoughts and behaviors (Franklin et al., 2017; Kiekens et al., 2018a), and rehospitalization following treatment (van Alphen et al., 2017). Rates of NSSI are highest among clinical populations (Glenn et al., 2017; Groschwitz et al., 2015), with half of adolescents and close to 10% of young adults who seek treatment reporting NSSI behavior in the past month (Millon et al., 2022; Ose et al., 2021). Recognizing its clinical importance and transdiagnostic nature across various mental disorders (i.e., emotional, eating, and personality disorders; Kiekens et al., 2018b, 2023a; Ose et al., 2021), the American Psychiatric Association recently included NSSI disorder as a condition requiring further research and added a diagnostic code for the presence of NSSI behavior in the DSM-5-TR (American Psychiatric Association, 2022).

Despite intensified efforts to better understand, predict, and prevent NSSI, the rate of NSSI has not meaningfully decreased over the past decades, with some work suggesting even increasing rates (Gillies et al., 2018; Wester et al., 2018). Supporting this, a recent meta-analysis of Randomized Controlled Trials (RCT) found no overall evidence that mental health treatments in the past 50 years significantly reduced the occurrence and frequency of self-injurious thoughts and behaviors (Fox, Huang et al., 2020). Although these findings paint a bleak picture of the present evidence base, some scholars argue that conventional treatments may not yet have reached their efficacy "ceiling", as their assessment and intervention modalities, which typically involve one or two weekly sessions, may not align with the dynamic nature of self-injurious thoughts and behaviors (Kleiman et al., 2022). For instance, recent work shows that NSSI thoughts and urges – which typically precede acts of self-injury – can fluctuate significantly across hours within one day, with NSSI behavior being most likely to occur in the late evening (Fitzpatrick et al., 2020; Kiekens et al., 2024). These findings underscore the potential clinical utility of integrating approaches that extend beyond the confines of the therapy room.

The clinical application of Ecological Momentary Assessment (EMA; also termed Experience Sampling; Myin-Germeys et al., 2024) might be particularly useful in this context (Bos et al., 2019; Kleiman et al., 2022). EMA is a structured diary technique that involves administering short questionnaires multiple times a day to users, nowadays, typically on mobile phones (Myin-Germeys et al., 2018; Stone & Shiffman, 1994). Researchers have advocated for the use of EMA in mental health treatment to facilitate person-centered care, enhance engagement, and promote self-management by enabling patients to monitor themselves (Bos et al., 2020; Kiekens et al., 2021; Myin-Germeys et al., 2024). However, to date, no study has investigated the extent to which individuals who self-injure experience clinical benefits and challenges associated with the use of EMA during treatment. In a recent pilot study, researchers used EMA in a small (n=17) specialized mental health care sample and found preliminary evidence that EMA facilitated patient reflection on their mental health, enhancing self-awareness and self-insight (Weermeijer et al., 2023; de Thurah et al., 2024). Similar findings were observed in clinical populations seeking treatment for bipolar disorder (n=20; Bos et al., 2020) and psychosis (Bell et al., 2017; de Thurah et al., 2023). Self-insight —the ability to recognize one’s mental health problems and become aware of their antecedents and consequences—has been associated with better global functioning, reduced psychopathology severity, and improved quality of life in clinical samples (Jennissen et al., 2018). To date, only one qualitative study has evaluated the feasibility and utility of EMA as an adjunct treatment tool for promoting self-insight among individuals who self-injure. Gromatsky et al. (2022) conducted semi-structured interviews with 34 U.S. Army veterans who used EMA three times daily for 28 days. Participants reported that self-monitoring facilitated insight into their intrapersonal experiences (e.g., emotions, cognitions, and behaviors), subsequently reducing their susceptibility to NSSI (Gromatsky et al., 2022).

A second potential clinical benefit of EMA is its capacity to enhance self-efficacy by stimulating self-management (Bos et al., 2019; de Thurah et al., 2024), defined as the belief in one’s ability to take effective actions toward achieving positive change (Bandura, 1997). Developing higher levels of self-efficacy has been shown to contribute to treatment gains and positive behavioral changes in mental health (Bandura, 1986, 1997) and is a protective factor for the onset of NSSI (Tatnell et al., 2014). Within the Cognitive-Emotional Model of NSSI (Hasking et al., 2017), self-efficacy to resist NSSI refers to an individual’s belief in their ability to implement actions and coping strategies to avoid engaging in NSSI (Hasking & Rose, 2016). EMA studies in both community and clinical samples have demonstrated that higher levels of self-efficacy to resist NSSI serve as a key protective factor against NSSI behavior (Kiekens et al., 2020, 2024). These findings underscore self-efficacy as a critical treatment target and suggest that individuals who self-injure may benefit from using EMA. However, the extent to which individuals seeking treatment report increased self-insight and self-efficacy in managing their emotions, cognitions, and behaviors, as well as NSSI-specific triggers, as a result of using EMA remains unclear.

At the same time, it is essential to consider that reflecting on one's emotions, thoughts, and behaviors multiple times per day might not only promote a sense of control but also evoke burden and emotional discomfort for some individuals (Bos et al., 2019; de Thurah et al., 2025; Weermeijer et al., 2023, 2024). Beep disturbance (also referred to in the field as burden) refers to the difficulty, inconvenience, or strain experienced when using EMA (Eisele, Kasanova et al., 2022; Eisele, Vachon et al., 2022; Kirtley, 2022; Rintala et al., 2019, 2021) whereas compliance is typically defined as the ratio of completed questionnaires to the total number of questionnaires received (Vachon et al., 2019). Recent findings from a large community sample of adolescents suggest that individuals with self-harm thoughts and behaviors have compliance rates similar to those with no history of self-harm but report slightly higher levels of beep disturbance (Kirtley et al., in press). However, there is also evidence that compliance rates may decrease over time (Rintala et al., 2019; Vachon et al., 2019). For example, the compliance of adolescents following discharge from psychiatric care for suicide risk decreased over a four-week assessment period, dropping from 87% in the first week to 45% in the final week (Glenn et al., 2022). While studies suggest no overall iatrogenic effects of repeated assessments on the risk of suicidal thoughts and behaviors (Coppersmith et al., 2022b; Glenn et al., 2022; Law et al., 2015), repeated EMA assessments may increase emotional distress for some individuals by making them acutely aware of their symptoms (Bos et al., 2019). For instance, Gromatsky et al. (2022) found that one-third of veterans using EMA reported initial discomfort related to reflecting on and reporting their mental health. These findings underscore the need to better understand the extent to which individuals who self-injure may experience benefits and challenges when using EMA as an adjunct tool in treatment. In doing so, it is critical to also explore whether specific patient characteristics can be prospectively associated with the subjective experience of using EMA in this vulnerable population.

### **The present study**

The present cohort study was designed to address these gaps in the literature. The objectives were to determine the extent to which individuals who self-injure and used EMA for 28 days subsequently: (1) report increased general self-insight and self-efficacy as well as NSSI-specific self-insight into NSSI triggers and self-efficacy to resist self-injury, and (2) experience beep disturbance and emotional discomfort. Additionally, we evaluated feasibility by considering compliance and explored whether sociodemographic variables (e.g., developmental age stages, educational level), mode of treatment, technological affinity, and clinical characteristics (e.g., psychological distress, NSSI severity characteristics, and outcome expectancies) predicted variation in the benefits and challenges of using EMA between patients. At the request of a reviewer, we also investigated momentary associations between beep disturbance, emotion dysregulation, and NSSI cognitions and behaviors.

## Methods

### **Design and sample**

Data for this study were drawn from the Detection of Acute rIsk for seLf-injurY (DAILY) project (see protocol paper; Kiekens et al., 2023a), an observational cohort study involving individuals seeking treatment who met the following inclusion criteria: (1) aged 15 to 39 years, (2) proficient in Dutch, (3) past-month NSSI urges and/or behaviors at intake, and (4) receiving inpatient or outpatient mental health treatment in the Flemish region of Belgium. Individuals with cognitive deficits that impeded comprehension of study materials were excluded. Patients were recruited via referral sampling from 21 mental health services, including nine inpatient, eight outpatient, and four hybrid-care services. Of these services, 11 services primarily addressed emotion dysregulation and mood-related conditions, five targeted social-emotional challenges associated with the transition from adolescence to emerging adulthood, three specialized in eating disorders, and two are private practices. Further information on the diagnostic characteristics of the participants is provided in Kiekens et al. (2024).

Eligible participants were informed about the study through mental health professionals and informational sessions held at these services. All patients completed a baseline assessment consisting of a questionnaire battery and a clinical interview. During this assessment, patients received an orientation and training on the EMA protocol using the m-Path app (Mestdagh et al., 2023). The 28-day EMA protocol commenced the following day and included: (1) six semi-random regular EMA surveys approximately every two waking hours between 10 AM and 9:30 PM, (2) three brief burst EMA surveys administered 5 to 10 minutes apart during the 30 minutes following reports of intense NSSI urges in the regular EMA surveys, and (3) event registrations of NSSI behaviors. Patients responded to EMA questions assessing emotions, cognitions, contextual information, and social appraisals. The primary EMA outcomes were NSSI cognitions (i.e., thoughts, urges, self-efficacy to resist NSSI) and NSSI behavior, while secondary EMA outcomes included the presence of disordered eating, substance use, and suicidal thoughts and behaviors. Several safety measures were implemented to safeguard patients during the monitoring period (Kiekens et al., 2023a). An automatic pop-up screen appeared at the end of any EMA survey in case a participant reported an intense urge to self-injure (score of ≥5 on a 7-point item), providing access to either general support resources or personal support options that patients provided during the baseline assessment. Furthermore, a safety protocol was activated when participants reported an intense urge to attempt suicide (response 6 or 7 on a 7-point scale), combined with low self-efficacy (responses 1-3 on a 7-point scale) to resist this urge. In such cases, a second pop-up prompted participants to provide additional information about the stressful situation they were in, and an automatic alert was sent to the study team. For inpatients, the clinical staff on duty was then notified by phone. For outpatients, a licensed clinical psychologist from the research team conducted a telephone-based risk assessment. If minors could not be reached and further action was needed, parents or legal guardians were also contacted when deemed appropriate. After the 28-day EMA period, a feedback survey was sent assessing participants' experiences. Participants and their treating clinicians also received a feedback report, summarizing patterns in emotions, cognitions, and harmful behaviors. Participants received financial compensation contingent on compliance, ranging from €20 to €100. The study received approval from the Ethics Committee Research UZ/KU Leuven. All participants provided written informed consent, with additional consent from parents or legal caregivers for minors (<18 years).

**Fig. 1:**
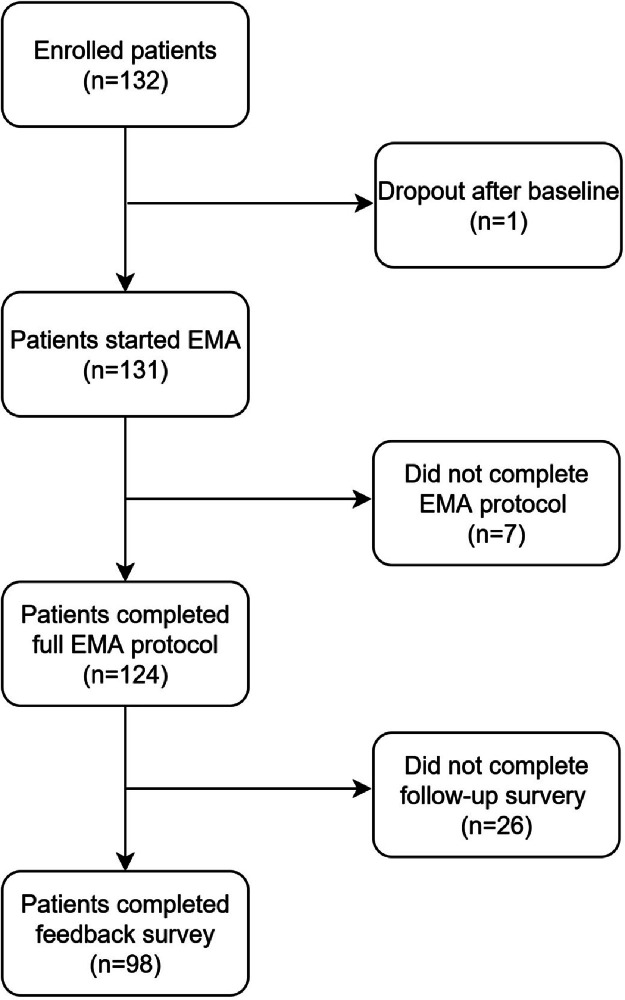
Patient flowchart of enrolment and analytical sample of the study.

Fig. 1 presents a flowchart of the cohort. A total of 132 patients were enrolled and completed the baseline assessments. One patient withdrew after baseline, and seven patients did not complete the 28-day EMA protocol, resulting in their exclusion from further analysis. Of the 124 patients who completed the 28-day EMA protocol, 98 patients completed the feedback questionnaire (response rate = 79.03%) and comprised the sample for the current study.

## Measures

### **Baseline measures**

**Sociodemographic information and mode of treatment.** Patients reported their age (in years), gender identity (female, male, or nonbinary), and highest educational attainment. Age was categorized into three developmental stages: adolescents (15-18 years), emerging adults (19-29 years), and adults (30-39 years). Education level was categorized into primary school, secondary school, or college/university (bachelor's, master's, or PhD). Patients also indicated the mode of treatment they were receiving, categorized as inpatient care, outpatient care, or hybrid care (a combination of both).

**Technological affinity**. The extent of patients' experience with and their affinity for technology was assessed using the Adapted Technology Affinity Scale (ATAS; Edison & Geissler, 2003). The ATAS consists of 11 items, each rated on a seven-point Likert scale ranging from 1 ("Totally disagree") to 7 ("Totally agree"), with a minimum and maximum range of 11-77. The scale demonstrated strong internal consistency (α = .90).

**Clinical characteristics.** General psychological distress in the past week, including symptoms of depression, anxiety, and stress, was assessed using the shortened Depression Anxiety Stress Scale (DASS-21; de Beurs et al., 2001; Lovibond, S.H & Lovibond, 1995). The DASS-21 includes 21 items rated on a four-point scale ranging from 0 ("Not at all" or "Never applicable") to 3 ("Very much" or "Mostly applicable"), with a minimum and maximum range of 0-63. The total score demonstrated good reliability in the present sample (α = .88).

The frequency of NSSI thoughts and behaviors was measured using the validated Self-Injurious Thoughts and Behaviors Interview (SITBI; Fox, Harris, et al., 2020; Nock et al., 2007). NSSI thoughts in the month prior to baseline were coded as follows: 1 = “1-4 times,” 2 = “5-20 times,” 3 = “21-50 times,” and 4 = " type="Periodical">51 times.” NSSI behaviors in the past month were coded as 1 = “0 times,” 2 = “1-4 times,” and 3 = “5 or more times.” NSSI outcome expectancies were assessed using the Non-Suicidal Self-Injury Expectancy Questionnaire (NEQ; Hasking & Boyes, 2018). The NEQ includes five subscales, each containing five items rated on a four-point scale ranging from 1 (“Extremely unlikely”) to 4 (“Extremely likely”), with a minimum and maximum range of 5-20. The subscales and their respective reliability in the present sample are: (1) affect regulation (α = .59), (2) negative social outcomes (α = .78), (3) communication (α = .70), (4) pain (α = .69), and (5) negative self-beliefs (α = .64). Finally, self-efficacy to resist NSSI in various situations and contexts in the coming weeks was assessed using an adapted version of the six-item Self-Efficacy to Avoid Suicidal Action scale (Kiekens et al., 2020). Items were rated on a ten-point scale ranging from 0 ("Very uncertain") to 9 ("Very certain"), with a minimum and maximum range of 0-54. The scale demonstrated good reliability in the present sample (α = .85).

### **EMA measures**

**NSSI thoughts and behavior.** EMA surveys assessed retrospective NSSI thoughts, momentary NSSI urges, and momentary perceived self-efficacy to resist NSSI using seven-point scales (Kiekens et al., 2023a). Participants rated the extent of NSSI thoughts ("*Since the last beep, have you considered deliberately hurting yourself without wanting to die?*") on a scale from 0 = “Not at all” to 6 = “Very much.” Momentary NSSI urges were assessed by asking, "*Right now, how strong is the urge to hurt yourself without wanting to die?*" with responses ranging from 0 = ”Absent” to 6 = ”Very strong.” Similarly, participants rated their momentary self-efficacy to resist NSSI (“*Right now, how confident are you that you can resist engaging in NSSI?*”) on a scale from 0 = ”Not at all” to 6 = ”Very confident.” Mean scores were computed for each patient for these three measures. NSSI behavior was assessed in two complimentary ways. First, participants were asked in each EMA survey whether they had engaged in NSSI ("*Since the last beep, have you deliberately hurt yourself without wanting to die [e.g., cut, scratched, or hit yourself]?*") using a dichotomous response format (no [0] or yes [1]). Second, participants could register their NSSI behavior directly within the EMA app. We computed the frequency of NSSI behavior across each patient's 28 days of EMA participation. The Dutch and English translations of the EMA items can be found in Supplementary Table 1.

**Compliance and beep disturbance**. Compliance was calculated as the number of answered regular EMA surveys divided by the total number of regular EMA surveys (i.e., 168 across 28 days). Beep disturbance was defined as the average response to the EMA-item “*I found this beep to be disturbing*”, which was rated on a seven-point scale ranging from 0 = “Not at all” to 6 = “Very much.” Higher scores reflected higher disruptiveness experienced during assessments. This question was assessed at the end of every questionnaire, with mean scores calculated for each patient across the 28-day EMA period.

**Emotion dysregulation.** During each assessment, participants rated the extent to which they experienced their emotions as overwhelming (“*Right now, my emotions are overwhelming me*”) on a scale ranging from 0 = “Not at all” to 6 = ”Very much.”

### Follow-up EMA feedback survey

Benefits and challenges of EMA were assessed during the EMA feedback survey using adapted questionnaires designed to capture self-reported changes related to using EMA. Items were framed as statements such as “*The self-monitoring made me ...*” or “*By what I learned about myself through the self-monitoring, ...*”. The cut-off scores for each scale were chosen to represent the minimum score that indicates a participant is expressing agreement on average with the questions of the scale. The scoring options varied per scale and are described below for each construct. The complete list of items is available in English in Supplementary Tables 2-6.

**Increased self-insight**. An adapted version of the Self-Reflection and Insight Scale (Grant et al., 2002) was used to assess improved awareness and insight into emotions, cognitions, and behaviors resulting from repeated EMA assessments. This measure consisted of 8 items rated on a six-point scale: 1 = "Strongly disagree," 2 = "Disagree," 3 = "Somewhat disagree," 4 = "Somewhat agree," 5 = "Agree," and 6 = "Strongly agree”. The scale demonstrated good internal reliability (α = 0.79). A cut-off score was set at a mean score of 4, reflecting agreement regarding increased self-insight due to using EMA.

**Increased self-efficacy.** An adapted version of a ten-item self-report questionnaire (Jerusalem & Schwarzer, 1992; Teeuw & Schwarzer, 1994) was used to measure the extent to which individuals felt better capable of solving problems, achieving goals, and managing unforeseen situations as a result of using EMA in treatment. Items were rated on a four-point scale ranging from 1 = "Not at all true," 2 = "Barely true," 3 = "Moderately true," to 4 = "Exactly true”. The scale demonstrated excellent internal reliability (α = 0.88). A cut-off score was set at a mean score of 3, indicating agreement regarding an increase in general self-efficacy due to using EMA.

**Increased NSSI-specific self-insight.** Five items adapted from the Self-Insight Scale (Grant et al., 2002) assessed participants’ increased understanding of triggers, urges, and behaviors related to NSSI, as well as awareness of related self-damaging thoughts and behaviors. Items were rated on a ten-point scale ranging from 1 = "Strongly disagree" to 10 = "Strongly agree”. The cut-off score was set to 6, with mean scores between 6 and 10 indicating agreement regarding an increase in NSSI-specific insight as a result of using EMA. The scale had excellent internal reliability (α = 0.88).

**Increased self-efficacy to resist NSSI.** Five items adapted from the Self-Efficacy Scale (Jerusalem & Schwarzer, 1992; Teeuw & Schwarzer, 1994) measured participants’ confidence in their ability to manage urges, avoid high-risk situations, and control NSSI-related thoughts and behaviors. Items were rated on a ten-point scale ranging from 1 = "Strongly disagree" to 10 = "Strongly agree”. The cut-off score was set to 6, with mean scores between 6 and 10 indicating agreement regarding an increase in NSSI-specific self-efficacy as a result of using EMA. The scale demonstrated good internal reliability (α = 0.85).

**Emotional discomfort.** Four self-developed items assessed participants' negative emotional reactions (e.g., feeling stressed, overwhelmed, tired) as a result of the repeated assessments. One item asked whether they enjoyed the use of EMA and was reverse-coded. Items were rated on a five-point scale, ranging from 1 = "Totally disagree" to 5 = "Totally agree”. The cut-off score was set to 4, with mean scores of 4 or higher indicating agreement with experiencing significant emotional discomfort related to the use of EMA. The scale had acceptable internal reliability (α = 0.71).

### **Open science practices**

The research aims and planned analyses for this study were post-registered on the Open Science Framework (OSF) after data collection, but before the data were accessed and analyses were conducted, using the registration template provided by Kirtley et al. (2021). The registration, deviation document, analysis code, and questionnaires from the DAILY study are publicly available on the OSF project page of this study: https://osf.io/gj3qm/. All EMA items from the DAILY project are available in the protocol paper (Kiekens et al., 2023a) and in the ESM Item Repository (Kirtley et al., 2024; www.esmitemrepository.com; dataset: “DAILY”).

### **Statistical analysis**

All analyses were conducted in RStudio (version 2024.4.0.735; Posit Team, 2024) and Mplus version 8.11 (Muthén & Muthén, 2017). Descriptive statistics, including response distributions, means, standard deviations, and proportions exceeding specified agreement cutoffs, were calculated to evaluate the extent to which individuals experienced benefits and challenges of using EMA. Bivariate correlations were estimated to assess associations between benefits and challenges. To assess the linear trend in compliance rates across the 28-day EMA period, a two-level random intercept multilevel linear model was estimated using the 'lme4′ package (Pinheiro et al., 2023). Linear regressions were used to evaluate prospective associations between baseline sociodemographic, technological affinity, and clinical characteristics and the benefits and challenges reported after 28 days of EMA. Bivariate models were run for each sociodemographic variable, and when significant, these were subsequently controlled for as confounding variables in models investigating other patient characteristics. Standardized beta coefficients are reported for independent continuous variables. All effects are evaluated with two-sided significance tests (α = 0.05) and their associated 95% confidence intervals (CIs).

Momentary associations between momentary beep disturbance, emotion dysregulation, and NSSI cognitions and behavior were investigated using Multilevel Vector Autoregressive (VAR) models within the (Residual) Dynamic Structural Equation Modeling (DSEM) framework (Asparouhov & Muthén, 2020; McNeish & Hamaker, 2020). Residual DSEM allows for a focus on contemporaneous associations within assessments, while standard DSEM captures temporal associations between assessments. Models were estimated using Bayesian methods with non-informative priors via Markov Chain Monte Carlo (MCMC) using Gibbs sampling. Momentary continuous variables were predicted using Bayesian linear regression, and NSSI behavior (i.e., binary yes-no variable) using Bayesian probit regression. To account for unequally spaced intervals due to missing data and random sampling within blocks, a two-hour transformed time interval was specified. Models included random intercepts and random slopes for autoregressive and cross-regressive effects, and residual variances of continuous variables were allowed to vary across individuals. To avoid bias from treating covariates as exogenous, all models included autoregressive effects for each momentary variable. Means and intercepts were allowed to covary. Unstandardized point estimates were obtained by taking each parameter’s median of the posterior distributions. Each model was estimated with at least 2,500 recorded iterations, using a thinning factor of 20. Model convergence was ensured by a potential scale reduction value close to 1. Statistical significance was defined by 95% credibility intervals that excluded 0, indicating a 95% probability that the effect differs from null.

## Results

### **Description of patient characteristics and EMA measures**

Table 1 summarizes the patient characteristics and EMA measures for the sample that completed the EMA feedback survey. The mean age was 23.4 years (*SD* = 5.37, range 15–39 years). Most patients were emerging adults (65.31%), identified as female (86.73%), and reported secondary education as their highest level of attainment (51.02%). Patients exhibited moderate technological affinity (*M* = 42.59, *SD* = 10.30) and elevated psychological distress in the week prior to intake (*M* = 36.21, *SD* = 9.63). Patients mostly expected NSSI to reduce negative affect (*M* = 14.10, *SD* = 2.43). NSSI behavior was reported an average of 9.97 times in the past month (*SD* = 18.61, range 0–150), with most patients (42.86%) indicating at baseline 21 to 50 NSSI thoughts in the past month. The majority (62.24%) of the sample who used EMA in their treatment were at least partially outpatients. Mean EMA intensity of patient-level averages for NSSI cognitions was 1.64 for retrospective NSSI thoughts (*SD* = 1.18, range 0–5.24), 1.59 for momentary urges (*SD* = 1.15, range 0–5.11), and 4.41 for momentary self-efficacy to resist NSSI (*SD* = 1.08, range 0.59–6.00). On average, patients engaged in NSSI 10.52 times during the EMA period (*SD* = 18.69, range 0–103).

**Table 1:** Patient characteristics at baseline and during EMA (n=98).

Sociodemographic Distribution	n(%) / Mean (SD, range)	Clinical Characteristics	n(%) / Mean (SD, range)
Developmental Stage:		General Psychological Distress	36.21 (9.63, 10-57)
Adolescents (15-18 years)	21 (21.43%)	NSSI outcome expectancies:	
Emerging adults (19-29 years)	64 (65.31%)	Affect regulation	14.10 (2.43, 7-19)
Adults (30-39 years)	13 (13.27%)	Negative Social Outcomes	13.55 (3.73, 5-19)
Gender:		Communication	9.30 (2.83, 5-16)
Female	85 (86.73%)	Pain	15.51 (2.77, 8-20)
Male	6 (6.12%)	Negative self-beliefs	15.33 (2.83,6-20)
Non-binary^a^	7 (7.14%)	Past-month NSSI Thoughts:	
Highest Educational Attainment:		1-4 times	8 (8.16%)
Primary education	24 (24.49%)	5-20 times	39 (39.80%)
Secondary education	50 (51.02%)	21-50 times	42 (42.86%)
University/college	24 (24.49%)	≥51 times	9 (9.18%)
Technological Affinity	42.59 (10.30, 9-63)	Past-month NSSI Behavior:	
Mode of Treatment		None	11 (11.22%)
Inpatient	37 (37.76%)	1-4 times	37 (37.76%)
Outpatient	37 (37.76%)	+5 times	50 (51.02%)
Hybrid (combination)	24 (24.49%)	Self-efficacy to resist NSSI	16.38 (10.53, 0-47)
			
NSSI during 28 days of EMA	n(%) / Mean (SD, range)		
Compliance	74.87 (18.78, 14.29-98.81)		
Disturbance (0-6 scale)	1.61 (1.25, 0-5.79)		
NSSI cognitions (0-6 scale):			
Retrospective NSSI thoughts	1.64 (1.18, 0-5.24)		
Momentary NSSI urges	1.59 (1.15, 0-5.11)		
Momentary self-efficacy to resist NSSI next hours	4.41 (1.08, 0.59-6.00)		
Frequency NSSI behavior	10.52 (18.69, 0-103)		

*Note*: ^a^ includes transgender male, transgender female, or self-defined. NSSI=Non-Suicidal Self-Injury, EMA=Ecological Momentary Assessment, SD=Standard Deviation.

### **Clinical benefits of using EMA during treatment**

Across the sample, 78.57 % reported that the use of EMA in treatment had at least one clinical benefit. The mean general self-insight score was 3.61 (*SD*=0.71), with mean scores ranging from 1.88 to 5.50 on a 1-6 scale (Table 2). The modal response was four (“Somewhat agree”) for all items except for the item 'The self-monitoring made me more aware of my feelings and thoughts' and 'The self-monitoring helped me to understand better who I am', where the modal response was five (“Agree”) and two (“Disagree”), respectively. One-third of patients (32.65%) agreed that using EMA during treatment increased their self-insight into their emotions, cognitions, and behavior. Conversely, two-thirds of patients (64.58%) reported increased insight into emotional-cognitive triggers and understanding of their NSSI cognitions and behaviors, with a mean score of 6.14 (*SD* = 1.57; range means 1.00–10.00) and a modal response of seven for all items on a 1-10 scale. Response distributions on individual items can be found in Supplementary Tables 2-3.

**Table 2:** Self-reported clinical benefits and challenges of using EMA during treatment.

	Mean (SD, range)	Agreement threshold for mean score^a^	Proportion above threshold	Sample size^b^
**Clinical Benefits of EMA use**				
General self-insight	3.61 (0.71, 1.88-5.50)	≥4^c^	32.65%	98
NSSI-specific self-insight	6.14 (1.57, 1.00-10.00)	≥6^d^	64.58%	96
General self-efficacy	2.29 (0.57, 1.00-4.00)	≥3^e^	9.28%	97
NSSI-specific self-efficacy	5.47 (1.77, 1.00-10.00)	≥6^d^	41.67%	96
**Clinical Challenges of EMA use**				
Compliance	74.87% (18.78, 14.29-98.81)	-	-	98
Disturbance	1.64 (1.18, 0.00-5.79)	-	-	98
Emotional discomfort	3.22 (0.75, 1.50-4.75)	≥4^f^	20.83%	96

*Note:*^a^ The cut-offs for the clinical benefits and challenges, which is the lowest scoring option that falls within the range of agreement for that scale. ^b^ number of non-missing responses varied between 96 and 98 across scales

c threshold range for mean score from somewhat agree (4) to strongly agree (6) on a 6-point scale with labels for each score ranging from 1 to 6

d threshold range for mean score in 6-10 range on a 10-point scale with anchors strongly disagree (1) to strongly agree (10)

e threshold range for mean score from moderately true (3) to exactly true (4) on a 4-point scale with labels for each score ranging from 1 to 4

f threshold range for mean score from agree (4) to totally agree (5) on a 5-point scale with labels for each score ranging from 1 to 5 (one item reverse-coded). Individual distributions of items can be consulted in supplementary materials.

Only 9.28% of patients agreed that the use of EMA increased their general self-efficacy, with a mean score of 2.29 (*SD* = 0.57; range 1.00–4.00) on a 4-point scale (Table 2). The modal response was two (“Barely true”) for all items, except for 'By what I learned about myself through the self-monitoring, I feel that I am better able to solve difficult problems if I try hard enough' and 'Whatever happens, what I have learned about myself through self-monitoring has given me more confidence I will get through it', for which the mode was 3 (”Moderately true”). A higher proportion of patients (41.67%) reported increased self-efficacy to resist NSSI due to the use of EMA during treatment, with a mean score of 5.47 (*SD* = 1.77; range means 1.00–10.00) and a mode response of 5-6 for all items on a 1-10 scale. Response distributions on individual items can be found in Supplementary Tables 4-5.

### **Clinical challenges of using EMA during treatment**

The mean EMA compliance rate for the 168 regular EMA surveys was 74.87% (*SD* = 18.78; 125.8 assessments on average per patient), ranging from 14.29% (24 assessments) to 98.81% (166 assessments). On average, patients completed thus 4.49 out of 6 regular surveys per day. Compliance declined significantly over time (*β* = *-* 0.43; SE = .05; *p* < .001). This decline translated to patients completing 4.84 surveys on average on the first day (80.71% compliance) compared to 4.14 surveys on the 28th day (69.02% compliance). Beep disturbance was generally rated moderately low (*M* = 1.64, *SD* = 1.18), with individual means for disruptiveness ranging substantially from 0.00 to 5.79 on a 0-6 scale. The mean score for emotional discomfort was 3.22 (*SD* = 0.75, range = 1.5–4.75) on a 1-5 scale. Response distributions of individual items revealed that most patients (58.33%) considered the use of EMA in treatment as a positive experience (Supplementary Table 6), while 34.38% were neutral, and 7.29% disagreed. Despite the overall positive experience, most patients found answering EMA questions tiring (56.25%), stress-inducing (54.17%), and at times emotionally overwhelming (63.54%). While the majority (78.13%) reported emotional discomfort across any of the four items, seven patients (7.29%) consistently reported EMA to be tiring, stressful, overwhelming, and not enjoyable.

### **Associations between benefits and challenges of EMA during treatment**

Table 3 reports associations between benefits and challenges. Small-to-moderate positive associations were observed between the range of benefits (range .30 ≤ *r* ≤ .68; *ps* ≤ .003) and between beep disturbance and emotional discomfort (*r* = .37, *p* < .001). Small-to-moderate negative associations were found between mean EMA compliance and emotional discomfort (*r* = -.29, *p* = .004), beep disturbance and general self-insight (*r* = -.21, *p* = .039), beep disturbance and general self-efficacy (*r* = -.33, *p* = .001), and beep disturbance and NSSI-specific self-efficacy (*r* = -.29, *p* = .004). While emotional discomfort was negatively associated with general self-insight (*r* = -.28, *p* = .006), there were no significant associations with any of the NSSI-specific outcomes (*p* ≥ .325*)*. Additionally, no association was observed between beep disturbance and compliance rates (*r* = -.07, *p* = .514).

**Table 3:** Associations between benefits and challenges of using EMA during treatment.

	General self-insight	NSSI^.^-specific self-insight	General self-efficacy	NSSI-specific self-efficacy	Mean EMA disturbance	EMA Compliance	Emotional discomfort
General self-insight	-	.39***	.48***	.30**	-.21*	.06	-.28**
NSSI-specific self-insight		-	.52***	.62***	-.16	.03	-.10
General self-efficacy			-	.68***	-.33**	.04	-.09
NSSI-specific self-efficacy				-	-.29**	.02	-.04
EMA disturbance					-	-.07	.37***
EMA compliance						-	-.29**
Emotional discomfort							-

*Note:* * *p*<0.05, ** *p*<0.01, *** *p*<0.001

At the request of a reviewer, we also examined momentary associations between beep disturbance, emotion dysregulation, and NSSI cognitions and behaviors. While no significant contemporaneous association was found between NSSI cognitions and beep disturbance (Supplementary Table 7), higher-than-usual emotion dysregulation predicted greater beep disturbance within the same assessment (*β* = 0.05, 95% CrI = 0.03–0.08). When examining associations over time, we observed bidirectional associations between emotion dysregulation and beep disturbance (Supplementary Table 8): higher-than-usual emotion dysregulation predicted greater beep disturbance at the next assessment (*β* = 0.04, 95% CrI = 0.02–0.06) and higher-than-usual beep disturbance also predicted an increase in how overwhelmed participants felt by their emotions (*β* = 0.04, 95% CrI = 0.01–0.06). Similarly, higher-than-usual retrospective NSSI thoughts predicted greater beep disturbance (*β* = 0.03, 95% CrI = 0.01–0.06) and higher-than-usual beep disturbance predicted more retrospective NSSI thoughts in the following two hours (*β* = 0.04, 95% CrI = 0.02–0.07). Higher-than-usual NSSI urges (*β* = 0.05, 95% CrI = 0.02–0.08) and lower self-efficacy to resist NSSI (*β* = -0.04, 95% CrI = -0.07 to -0.02) also predicted greater beep disturbance at the next assessment, but the reverse direction was not observed (Supplementary Table 9). When controlling for momentary emotion dysregulation, NSSI thoughts no longer predicted beep disturbance (*β* = 0.02, 95% CrI = 0.00–0.04). No temporal associations were observed between beep disturbance and NSSI behavior between assessments (Supplementary Table 10).

### **Patient characteristics as predictors of benefits and challenges of EMA during treatment**

Finally, we investigated whether sociodemographic characteristics, mode of treatment, technological affinity, and clinical characteristics predicted the benefits and challenges of EMA (Tables 4 and 5). Females reported a higher compliance rate than males (*β* = 16.68, p = .036) and adults reported less beep disturbance than adolescents (*β* = -0.97, p = .026). Controlling for these sociodemographics, few significant associations were found for clinical characteristics (Table 5).

**Table 4:** Sociodemographic characteristics predicting benefits and challenges of using EMA during treatment.

Sociodemographic characteristics	Benefits of EMA				Challenges of EMA		
	General insight	NSSI-specific insight	General Self-efficacy	NSSI-specific self-efficacy	Compliance	Disturbance	Emotional discomfort
Gender:	β (CI 95%)	β (CI 95%)	β (CI 95%)	β (CI 95%)	β (CI 95%)	β (CI 95%)	β (CI 95%)
	Reference	Reference	Reference	Reference	Reference	Reference	Reference
Female							
Male	-0.58 (-1.17; 0.01)	-0.77 (-2.21; 0.67)	-0.24 (-0.77; 0.28)	-0.38 (-2.00;1.24)	**-16.68** **(-32.23; -1.14)**	-0.22 (-1.28; 0.83)	-0.02 (-0.71; 0.67)
Other	0.13 (-0.42; 0.68)	-0.38 (-1.61; 0.85)	0.00 (-0.45; 0.44)	-0.68 (-2.07; 0.70)	-1.60 (-16.07; 12.86)	0.12 (-0.86; 1.10)	0.07 (-0.52; 0.66)
Developmental Stage:	Reference	Reference	Reference	Reference	Reference	Reference	Reference
Adolescents (15-18 years)							
Emerging adults (19-29 years)	0.15 (-0.20; 0.51)	0.19 (-0.60; 0.98)	0.17 (-0.12; 0.45)	0.65 (-0.24; 1.53)	-0.25 (-9.66; 9.17)	-0.56 (-1.17; 0.05)	0.20 (-0.17; -0.57)
Adults (30-39 years)	0.29 (-0.21; 0.79)	0.68 (-0.43; 1.78)	0.30 (-0.09; 0.70)	0.80 (-0.44; 2.03)	5.82 (-7.39; 19.03)	**-0.97** **(-1.83; -0.11)**	-0.20 (-0.72; 0.33)
Highest Educational Attainment:	Reference	Reference	Reference	Reference	Reference	Reference	Reference
Primary education							
Secondary education	0.10 (-0.25; 0.46)	-0.26 (-1.05; 0.52)	0.13 (-0.15; 0.42)	0.19 (-0.70; 1.07)	-2.6 (-11.9; 6.70)	-0.52 (-1.13; 0.09)	0.18 (-0.20; 0.55)
University/college	0.12 (-0.29; 0.54)	0.02 (-0.89; 0.92)	0.05 (-0.28; 0.37)	0.27 (-0.75; 1.29)	2.16 (-8.65; 12.97)	-0.65 (-1.35; 0.06)	0.03 (-0.40; 0.46)

Note: Each cel shows the results of a separate linear regression with the variable in the row as predictor and the variable in the column as the outcome. Significant effects (*p*<0.05) are bolded.

**Table 5:** Mode of treatment, technological affinity, and clinical characteristics predicting benefits and challenges of using EMA during treatment.

Predictors		Benefits of EMA			Challenges of EMA		
	General insight	NSSI-specific insight	General Self-efficacy	NSSI-specific self-efficacy	Compliance	Disturbance	Emotional discomfort
	β (CI 95%)	β (CI 95%)	β (CI 95%)	β (CI 95%)	β (CI 95%)	β (CI 95%)	β (CI 95%)
Technological Affinity	0.09 (-0.05; 0.24)	0.06 (-0.27; 0.38)	0.03 (-0.09; 0.14)	-0.14 (-0.50; 0.22)	-1.66 (-5.54; 2.22)	-0.14 (-0.38; 0.11)	-0.01 (-0.17; 0.14)
Mode of treatment:	Reference	Reference	Reference	Reference	Reference	Reference	Reference
Inpatient							
Outpatient	0.10 (-0.23; 0.44)	0.60 (-0.13; 1.34)	0.18 (-0.08; 0.45)	0.16 (-0.67 1.0)	1.66 (-6.91; 10.24)	-0.28 (-0.83; 0.28)	-0.22 (-0.57; 0.12)
Hybrid (combination)	- 0.08 (-0.45; 0.29)	0.15 (-0.66; 0.97)	0.06 (-0.23; 0.36)	-0.29 (-1.21; 0.63)	-4.88 (-14.55; 4.80)	**0.76** (0.14; 1.38)*	0.24 (-0.15; 0.62)
Clinical characteristics							
General Psychological Distress	-0.01 (-0.16; 0.13)	**0.39** (0.08;0.70)*	-0.01 (-0.13; 0.10)	-0.01 (-0.37; 0.34)	**-4.89** (-8.53; -1.25)*	**0.36** (0.12; 0.60)**	**0.15** (0.003; 0.30)*
NSSI outcome expectancies:							
Affect regulation	0.05 (-0.09; 0.19)	-0.04 (-0.37; 0.28)	-0.05 (-0.16; 0.07)	-0.23 (-0.59; 0.13)	-3.40 (-7.09; 0.30)	-0.23 (-0.47; 0.02)	-0.09 (-0.24; 0.06)
Negative Social Outcomes	-0.03 (-0.18; 0.11)	0.06 (-0.26; 0.38)	0.01 (-0.11; 0.12)	-0.15 (-0.51; 0.22)	-2.90 (-5.94; 1.75)	**0.36** (0.12; 0.60)**	**0.16** (0.01; 0.31)*
Communication	0.09 (-0.06; 0.23)	**0.32** (0.01; 0.64)*	0.10 (-0.01; 0.21)	0.32 (-0.04; 0.67)	-2.38 (-6.11; 1.36)	0.08 (-0.17; 0.32)	0.02 (-0.13; 0.17)
Pain	**-0.19** (-0.33; -0.06)**	-0.14 (-0.46; 0.18)	-0.05 (-0.16; 0.07)	0.17 (-0.19; 0.53)	3.53 (-0.33; 7.38)	-0.06 (-0.31; 0.19)	0.01 (-0.15; 0.16)
Negative self-beliefs	0.06 (-0.08; 0.21)	0.30 (-0.02; 0.63)	0.05 (-0.07; 0.17)	0.10 (-0.27; 0.47)	0.91 (-2.95; 4.77)	-0.02 (-0.27; 0.23)	0.06 (-0.10; 0.21)
Past-month NSSI Thoughts:	Reference	Reference	Reference	Reference	Reference	Reference	Reference
1-4 times							
5-20 times	0.05 (-0.50; 0.61)	-0.37 (-1.59; 0.85)	-0.02 (-0.46; 0.42)	-0.26 (-1.58; 1.07)	-2.21 (-16.96; 12.55)	0.45 (-0.49; 1.38)	-0.31 (-0.88; 0.27)
21-50 times	0.06 (-0. 50; 0.61)	-0.83 (-2.03; 0.38)	-0.20 (-0.64; 0.24)	-1.26 (-2.58; 0.05)	-4.61 (-19.04; 9.81)	**0.96** (0.02; 1.90)*	0.09 (-0.48; 0.65)
≥51 times	-0.11 (-0.80; 0.59)	-0.59 (-2.11; 0.93)	-0.16 (-0.70; 0.39)	-1.11 (-2.77; 0.54)	-4.36 (-22.55; 13.83)	0.75 (-0.43; 1.93)	-0.12 (-0.83; 0.60)
Past-month NSSI Behavior:	Reference	Reference	Reference	Reference	Reference	Reference	Reference
None							
1-4 times	0.01 (-0.48; 0.50)	0.65 (-0.41; 1.72)	-0.04 (-0.42; 0.35)	-0.50 (-1.69; 0.69)	-6.15 (-18;66; 6.36)	0.43 (-0.40; 1.27)	0.004 (-0.48; 0.49)
+5 times	-0.14 (-0.61; 0.33)	-0.09 (-1.11; 0.94)	-0.20 (-0.58; 0.17)	**-1.19** (-2.34; -0.05)*	-11.79 (-23.89; 0.32)	0.66 (-0.16; 1.47)	0.55 (0.09; 1.02)*
Self-efficacy to resist NSSI	0.07 (-0.08; 0.21)	0.10 (-0.24; 0.43)	0.07 (-0.05; 0.19)	0.33 (-0.04; 0.70)	0.71 (-3.16; 4.58)	-0.09 (-0.33; 0.16)	-0.08 (-0.24; 0.08)

*Note*: Each cell shows the results of a separate linear regression with the variable in the row as a predictor and the variable in the column as the outcome, controlling for relevant sociodemographic predictors. Significant effects are bolded. * *p*<0.05, ** *p*<0.01.

Patients receiving a combination of in and out-patient care reported higher beep disturbance (Adjusted *β* = 0.76, p = .018) compared with those receiving inpatient care. Higher levels of general psychological distress at intake were associated with lower compliance (Adjusted *β* = -4.89, *p* = .009) and increased emotional discomfort (*β* = 0.15, *p* = .048) and beep disturbance (*β* = 0.36, *p* = .004), but also with greater increases in NSSI-specific self-insight (*β* = 0.39, *p* = .011). Patients who engaged in five or more NSSI behaviors in the month prior to starting the EMA protocol reported significantly greater emotional discomfort (*β* = 0.55, *p* = .02) and fewer increases in NSSI-specific self-efficacy (*β* = -1.19, *p* = .041) than those without past-month NSSI behavior. Individuals who experienced 21 to 50 NSSI thoughts in the month prior to starting the EMA also reported greater disturbance (Adjusted *β* = 0.96, *p* = .045) than those with 1-4 NSSI thoughts. Finally, patients who expected NSSI to serve a communicative function reported greater increases in NSSI-specific self-insight (*β* = 0.32, *p* = .044), whereas patients who expected NSSI to lead to negative social outcomes reported greater disturbance (Adjusted *β* = 0.36, *p* = .004) and emotional discomfort (*β* = 0.16 *p* = .038) of using EMA in treatment.

## Discussion

This clinical cohort study evaluated the self-reported clinical benefits and challenges associated with the use of EMA among patients who report NSSI at treatment intake. Four key findings emerged that warrant further discussion. First, four out of five (78.57%) patients retrospectively reported experiencing benefits from using EMA, with a nearly equal amount of participants (78.13%) also reporting some level of emotional discomfort related to using EMA. Second, the perceived benefits were higher for NSSI-specific self-insight and self-efficacy compared to general self-insight and self-efficacy. Third, patients reported greater momentary beep disturbance when they felt more overwhelmed by their emotions, and we observed small short-term increases in NSSI thoughts and emotion dysregulation following EMA surveys that were perceived as more disturbing. Fourth, few patient characteristics were consistently associated with self-reported outcomes. Each of these findings has clinical implications and will be discussed.

First, approximately one-third of patients reported increased general self-insight, suggesting that frequent self-monitoring and reflection involved in EMA may create awareness and help individuals better understand their emotions, cognitions, and behaviors in the context of daily life (Bos et al., 2019; de Thurah et al., 2024; Spangenberg et al., 2025; Weermeijer et al., 2023). Repeated self-monitoring has been shown to increase awareness of emotional and cognitive states (Kauer et al., 2012), which patients who self-injure may tend to suppress or avoid (Angelakis & Gooding, 2021; Spitzen et al., 2022). However, less than one in ten patients reported improvements in general self-efficacy. This discrepancy suggests that while EMA might increase self-insight among patients during treatment, translating this into a broader sense of capability or confidence across diverse contexts might require additional interventions and social support (de Thurah et al., 2024; Rippon et al., 2024).

Second, self-reported NSSI-specific outcomes were remarkably higher than those on general outcomes, indicating that using EMA in this population may specifically facilitate better insight and understanding of situations, emotions, and cognitions that trigger NSSI and comorbid emotion-regulating behaviors. Subsequently, this increased insight may help patients who engage in NSSI to become aware of situations that increase the risk for self-injury, allowing them to deploy more adaptive coping mechanisms, thereby increasing their self-efficacy to resist NSSI (Hasking et al., 2017). These findings are consistent with prior qualitative findings (Ball et al., 2025; Gromatsky et al., 2022; Spangenberg et al., 2025), which highlight the importance of personalizing the content of EMA surveys so that relevant experiences are assessed, increasing the clinical usability, and the patient’s engagement with EMA as a monitoring tool during treatment.

Third, the current study also highlights challenges associated with the use of EMA among patients who self-injure. Consistent with previous studies in clinical samples (Glenn et al., 2022; Kivelä et al., 2024; Rintala et al., 2019), we found that compliance decreased over time. Our finding aligns with recent findings from Ball et al. (2025), who investigated the engagement with EMA across five weeks in 100 hospitalized adults with suicidal thoughts and behaviors, and found that while EMA engagement was relatively high during hospitalization (66%), it dropped post-discharge (54%). While beep disturbance was rated relatively low across the sample, it was somewhat higher among adolescents. This may reflect greater clinical severity of adolescents who self-injure and seek treatment (Kiekens et al., 2024), as well as ongoing development of self-regulatory and executive functioning skills, which can heighten the perceived burden of EMA prompts (Ferguson et al., 2021). Moreover, prior research has shown that adolescents report greater disturbance when they are with peers or friends compared to family members, suggesting that social context may further contribute to the experience of burden (van Roekel et al., 2019). To address this, future research could increase the response flexibility (e.g., temporary “suspend” options) and tailor EMA content to adolescents’ developmental needs and contexts (van Roekel et al., 2019). Although most patients retrospectively considered the use of EMA positive overall, they also reported experiencing emotional discomfort as a result of engaging in EMA. These findings align with a recent study of 58 adults with past-year suicidal thoughts and behaviors (Kivelä et al., 2024). Of note, in the current study, patients experiencing emotional discomfort reported lower EMA compliance and higher disturbance. While they were less likely to report that using EMA led to increased general self-insight, they reported benefitting equally in terms of NSSI-specific outcomes.

Previous studies have demonstrated that EMA does not lead to systematic mood reactivity (Kivelä et al., 2024), with greater assessment frequency not being associated with increased suicidal thoughts (Coppersmith et al., 2022b). With respect to NSSI, prior work from our group using data from the DAILY project revealed no systematic changes in the intensity of NSSI thoughts and NSSI urges over time but showed an increase in self-efficacy to resist NSSI and a decrease in NSSI behavior across the four weeks of EMA (Kiekens et al., 2024). While these changes may reflect EMA effects, treatment effects, or both, some patients in the current study retrospectively reported feeling overwhelmed or stressed by completing EMA. Our within-person analyses revealed that when individuals felt more overwhelmed by their emotions, they found it more taxing to complete not only the current but also the subsequent EMA survey. Although we did not observe contemporaneous associations with NSSI cognitions within the same assessment, recent findings by Kirtley et al., in press showed that community adolescents with more intense self-harm thoughts also reported higher levels of beep disturbance. Interestingly, while those authors did not find temporal associations in their community sample, we identified small but significant bidirectional relationships between emotion dysregulation and NSSI thoughts, on the one hand, and beep disturbance, on the other hand. Importantly, while increased beep disturbance predicted subsequent increases in emotion dysregulation and retrospectively reported NSSI thoughts two hours later, this was not observed for NSSI urges, self-efficacy to resist NSSI, or NSSI behavior.

This pattern of findings suggests that monitoring oneself with EMA—particularly during moments when it is perceived as more burdensome and effortful—may subsequently lead to short-term experiential changes in emotion and cognition, without necessarily increasing iatrogenic action tendencies or the risk of NSSI behavior. These results align with those of Spangenberg et al. (2025), who reported that reactivity can be both positive (e.g., increased introspection and self-reflection) and burdensome (e.g., cognitively effortful and heightened emotional awareness). Yet, such emotional discomfort can serve an important therapeutic purpose, much like in exposure-based interventions. By becoming more aware of emotional states—rather than suppressing or avoiding them—patients can learn that emotions are functional and can be managed without necessitating harmful action, even if this initially leads to some emotional discomfort. This is an important treatment goal in existing NSSI programs such as the Cutting Down Program (Kaess et al., 2020; Rockstroh et al., 2023), Emotion Regulation Individual Therapy (Bjureberg et al., 2023), Dialectical Behavioral Therapy (Linehan, 2015), and Treatment for Self-Injurious Behaviors (Andover et al., 2020). Integrating EMA in these evidence-based programs may foster greater insight into the contingencies of NSSI and help facilitate self-efficacy to resist NSSI, potentially further enhancing the effectiveness of these treatments.

Fourth and final, few patient characteristics consistently predicted the experienced benefits and challenges of EMA during treatment. Individuals with higher baseline psychological distress in the month before EMA-use reported greater increases in NSSI-specific self-insight but also experienced more emotional discomfort, greater EMA-related disturbance, and lower compliance rates. This partially aligns with previous findings (Kivelä et al., 2024), showing that individuals with greater psychopathology are more likely to report emotional discomfort. Notably, while these individuals may have more to gain from EMA, they also experience a heightened emotional burden, underscoring the need for clinician involvement to help patients interpret their EMA data and provide tailored support throughout its use (de Thurah et al., 2024; Weermeijer et al., 2023). This may be particularly important for patients who believe that NSSI communicates distress to others or leads to negative social consequences (e.g., rejection), as these beliefs were linked to increased NSSI-specific self-insight and greater EMA-related emotional discomfort and disturbance. Similarly, patients who engaged in more frequent NSSI in the month prior to intake reported fewer increases in NSSI-specific self-efficacy and greater emotional discomfort as a result of EMA, highlighting the need for targeted support in this group. For these individuals, NSSI may be more entrenched, and building self-efficacy to resist NSSI might require the integration of Ecological Momentary Interventions or just-in-time-adaptive interventions alongside EMA (Coppersmith et al., 2022a; Dao et al., 2021). Future work should explore the perspectives of patients and clinicians regarding the utility and scope of these digital tools as potential adjuncts to conventional therapy.

### Limitations and future research

This study has several important limitations that should be considered when interpreting the results. First, participants’ experiences with EMA were assessed at the end of the study. Although the items were specifically designed to capture the benefits and challenges of EMA following its use for 28 days, the retrospective nature of these assessments introduces the possibility of recall bias. Additionally, because patients discussed their EMA data with mental health professionals, future work with pre-post assessments is needed to disentangle the specific effects of EMA from treatment-related changes due to discussing EMA feedback, as well as a potential social desirability bias. Such efforts would benefit from the development of a questionnaire designed to assess individuals' awareness and insight into the situational, emotional, and cognitive factors that increase their risk for NSSI in daily life. Simultaneously, future research should also examine how clinician characteristics and their perspective determine the utility of EMA as a therapeutic assessment tool.

Second, the composition of the sample limits the generalizability of our findings to males, non-binary individuals, and early adolescents (aged 10–14 years) seeking treatment, warranting further research in these populations. Additionally, findings may not generalize to minority populations or to settings with different cultural norms or legal frameworks around self-injury, particularly those where mandatory reporting to authorities is required (Hoelscher et al., 2025). Future research should examine how EMA is received and experienced across diverse sociocultural care contexts and consider culturally sensitive adaptations to enhance its clinical relevance and acceptability. Third, while the average EMA compliance rate of 74.87% is good and may suggest acceptability, it may not readily translate to routine clinical practice, where there will be no financial reimbursement (Ball et al., 2025). Future studies should explore strategies to optimize EMA protocols. For example, using single-item affect measures alongside adaptable beep schedules might increase engagement (Cloos et al., 2023; Eisele, Vachon et al., 2022; Vachon et al., 2019) as well as benefits from non-monetary incentives (e.g. personalized feedback reports, in-app gamification, and regular clinician check-ins) shown to maintain adherence over longer EMA periods (Mink et al., 2025; Vachon et al., 2019). An EMA feedback report, as was provided to clinicians to discuss with patients, may also sustain or improve compliance and enhance the perceived benefits of EMA. These strategies could enhance patients’ future engagement with EMA, and ensure that the processes monitored and the interpretation thereof are clinically relevant and meaningful.

Fourth, we reported on the general experiences of EMA among individuals seeking treatment as usual across various settings. Future studies should explore both the benefits and challenges of using EMA at different stages of the treatment process (Mink et al., 2025). For example, the TheraNet Project (Hall et al., 2025) examined therapists' experiences with pre-treatment EMA-based personalized feedback. Therapists reported that such feedback may support case conceptualization and therapy planning (Hall et al., 2025), but further research is needed to investigate how EMA can be integrated into the therapeutic processes of specific treatments (Mink et al., 2025). With regard to the domains assessed, several domains may be especially relevant for individuals who self-injure, including affective states and emotion regulation (Kiekens et al., 2020; Kuehn et al., 2022), negative cognitive styles (e.g., self-critical or repetitive thinking; Hughes et al., 2019; Robillard et al., 2025), urges and cognitions related to NSSI (Fitzpatrick et al., 2020; Kiekens et al., 2024), as well as key contextual factors such as interpersonal processes and conflict (Janssens et al., 2024; Turner et al., 2016), daily stressors (Haliczer & Dixon-Gordon, 2023; Kuburi et al., 2024), and sleep (Burke et al., 2022). Previously used items to assess these domains can be consulted in the ESM Item Repository (Kirtley et al., 2024). Assessing these domains—especially when collaboratively selected with the patient to tailor to individual needs—may support therapeutic dialogue about treatment goals. Recent clinical EMA platforms (e.g., Personalizes Treatment by Real-time Assessment (PETRA) and m-Path; Bos et al., 2022; Mestdagh et al., 2023) could help facilitate the co-construction of personalized EMA diaries.

Fifth, while the present study focused on self-reported benefits and challenges of EMA, future research should explore the momentary experience of these benefits and burdens, as well as between-person differences and potential predictors of how they are perceived (e.g., treatment history and level of integration in ongoing therapy). Additionally, examining predictors of prompt-level missingness could help refine EMA protocols to enhance engagement and improve data quality, particularly across diverse clinical settings (see Jacobucci et al., 2024). Finally, this study investigated the self-reported benefits and challenges of EMA among patients who self-injure quantitatively; further qualitative research is needed to increase understanding of the psychological processes underlying these findings. Exploring patients' experiences may reveal nuances not captured by quantitative measures, offering insight into the mechanisms through which EMA facilitates therapeutic engagement outside the therapy room. Addressing these directions will be important before evaluating the utility and efficacy of EMA in resource-intensive randomized controlled trials.

## Conclusion

This study provides novel information about the self-reported benefits and challenges of using EMA among patients who engage in NSSI and are seeking treatment. Four out of five patients who used EMA for 28 days reported benefits, particularly increased insight into the emotional and cognitive states that heighten NSSI risk. Only 7.29% consistently found EMA tiring, stressful, overwhelming, and unenjoyable. Although most patients reported some level of emotional discomfort, this was not consistently related to the perceived benefits. Using EMA may support the development of NSSI-specific self-insight and self-efficacy outside the therapy room. Future research is needed to determine the working mechanisms behind the reported clinical benefits, how they can be optimized, and how EMA-derived insights can be meaningfully integrated into therapeutic practice.

## Funding

This study is supported by the Research Foundation Flanders (12ZZM21N/1204924N; GK). RAB, LdT, LU and JDW are funded by the European Union's Horizon 2020 Research and Innovation Programme under (945263; IMG). JRB was supported by a Marie Skłodowska-Curie fellowship (101063326). During this work, OJK received support from a KU Leuven C+ grant (CPLUS/24/009).

## Declaration of competing interest

The authors have no competing interests to declare. The funding source had no role in the design and conduct of the study; collection, management, analysis, interpretation; preparation, review, or approval of the manuscript; and decision to submit the manuscript for publication.

## Appendix Supplementary materials

Image, application 1

## References

[bib0001] American Psychiatric Association (2022).

[bib0002] Andover M.S., Schatten H.T., Holman C.S., Miller I.W. (2020). Moderators of treatment response to an intervention for nonsuicidal self-injury in young adults. Journal of Consulting and Clinical Psychology.

[bib0003] Angelakis I., Gooding P. (2021). Experiential avoidance in non-suicidal self-injury and suicide experiences: A systematic review and meta-analysis. Suicide and Life-Threatening Behavior.

[bib0004] Asparouhov T., Muthén B. (2020). Comparison of models for the analysis of intensive longitudinal data. Structural Equation Modeling: A Multidisciplinary Journal.

[bib0005] Ball M.I., Fishbein N.S., Ramlal N., Hu N., Maimone J.S., Bear A., Kleiman E.M., Stein M.B., Nock M.K., Bentley K.H. (2025). Engagement in ecological momentary assessment of suicidal thoughts and behaviors: A mixed methods study. Behavior Therapy.

[bib0006] Bandura A. (1986). Social foundations of thought and action. Englewood Cliffs, NJ.

[bib0007] Bandura A. (1997).

[bib0008] Bell I.H., Lim M.H., Rossell S.L., Thomas N. (2017). Ecological momentary assessment and intervention in the treatment of psychotic disorders: A systematic review. Psychiatric Services.

[bib0009] Bjureberg J., Ojala O., Hesser H., Häbel H., Sahlin H., Gratz K.L., Hellner C. (2023). Effect of internet-delivered emotion regulation individual therapy for adolescents with nonsuicidal self-injury disorder: A randomized clinical trial. JAMA Network Open.

[bib0010] Bos F.M., Snippe E., Bruggeman R., Doornbos B., Wichers M., van der Krieke L. (2020). Recommendations for the use of long-term experience sampling in bipolar disorder care: A qualitative study of patient and clinician experiences. International Journal of Bipolar Disorders.

[bib0011] Bos F.M., Snippe E., Bruggeman R., Wichers M., van der Krieke L. (2019). Insights of patients and clinicians on the promise of the experience sampling method for psychiatric care. Psychiatric Services.

[bib0012] Bos F.M., Von Klipstein L., Emerencia A.C., Veermans E., Verhage T., Snippe E., Riese H. (2022). A web-based application for personalized ecological momentary assessment in psychiatric care: User-centered development of the PETRA application. JMIR Mental Health.

[bib0013] Burke T.A., Hamilton J.L., Seigel D., Kautz M., Liu R.T., Alloy L.B., Barker D.H. (2022). Sleep irregularity and nonsuicidal self-injurious urges and behaviors. Sleep.

[bib0014] Cloos L., Ceulemans E., Kuppens P. (2023). Development, validation, and comparison of self-report measures for positive and negative affect in intensive longitudinal research. Psychological Assessment.

[bib0015] Coppersmith D.D., Dempsey W., Kleiman E.M., Bentley K.H., Murphy S.A., Nock M.K. (2022). Just-in-time adaptive interventions for suicide prevention: Promise, challenges, and future directions. Psychiatry.

[bib0016] Coppersmith D.D.L., Fortgang R.G., Kleiman E.M., Millner A.J., Yeager A.L., Mair P., Nock M.K. (2022). Effect of frequent assessment of suicidal thinking on its incidence and severity: High-resolution real-time monitoring study. The British Journal of Psychiatry.

[bib0017] Dao K.P., De Cocker K., Tong H.L., Kocaballi A.B., Chow C., Laranjo L. (2021). Smartphone-delivered ecological momentary interventions based on ecological momentary assessments to promote health behaviors: Systematic review and adapted checklist for reporting ecological momentary assessment and intervention studies. JMIR mHealth and uHealth.

[bib0018] de Beurs E., Van Dyck R., Marquenie L.A., Lange A., Blonk R.W. (2001). De DASS: een vragenlijst voor het meten van depressie, angst en stress. Gedragstherapie.

[bib0019] de Thurah L., Kiekens G., Sips R., Teixeira A., Kasanova Z., Myin-Germeys I. (2023). Using Experience Sampling Methods to support clinical management of psychosis: The perspective of people with lived experience. Psychiatry Research.

[bib0020] de Thurah L., Kiekens G., Weermeijer J., Uyttebroek L., Wampers M., Bonnier R., Myin-Germeys I. (2025). Understanding appropriation of digital self-monitoring tools in mental health care: Qualitative analysis. JMIR Human Factors.

[bib0021] de Thurah, L., Weermeijer, J. D. M., Uyttebroek, L., Wampers, M., Bonnier, R., Myin-Germeys, I., & Kiekens, G. (2024, September 27). Can Experience Sampling Self-monitoring tools promote the activation of clients in mental healthcare? A qualitative study. Preprint: 10.31219/osf.io/2mxs7. 40132028

[bib0022] Edison S.W., Geissler G.L. (2003). Measuring attitudes towards general technology: Antecedents, hypotheses and scale development. Journal of Targeting, Measurement and Analysis for Marketing.

[bib0023] Eisele G., Kasanova Z., Houben M. (2022). https://www.kuleuven.be/samenwerking/real/real-book.

[bib0024] Eisele G., Vachon H., Lafit G., Kuppens P., Houben M., Myin-Germeys I., Viechtbauer W. (2022). The effects of sampling frequency and questionnaire length on perceived burden, compliance, and careless responding in experience sampling data in a student population. Assessment.

[bib0025] Ferguson H.J., Brunsdon V.E.A., Bradford E.E.F. (2021). The developmental trajectories of executive function from adolescence to old age. Scientific Reports.

[bib0026] Fitzpatrick S., Kranzler A., Fehling K., Lindqvist J., Selby E.A. (2020). Investigating the role of the intensity and duration of self-injury thoughts in self-injury with ecological momentary assessment. Psychiatry Research.

[bib0027] Fox K.R., Harris J.A., Wang S.B., Millner A.J., Deming C.A., Nock M.K. (2020). Self-injurious thoughts and behaviors interview—revised: Development, reliability, and validity. Psychological Assessment.

[bib0028] Fox K.R., Huang X., Guzmán E.M., Funsch K.M., Cha C.B., Ribeiro J.D., Franklin J.C. (2020). Interventions for suicide and self-injury: A meta-analysis of randomized controlled trials across nearly 50 years of research. Psychological Bulletin.

[bib0029] Franklin J.C., Ribeiro J.D., Fox K.R., Bentley K.H., Kleiman E.M., Huang X., Musacchio K.M., Jaroszewski A.C., Chang B.P., Nock M.K. (2017). Risk factors for suicidal thoughts and behaviors: A meta-analysis of 50 years of research. Psychological Bulletin.

[bib0030] Gillies D., Christou M.A., Dixon A.C., Featherston O.J., Rapti I., Garcia-Anguita A., Villasis-Keever M., Reebye P., Christou E., Al Kabir N., Christou P.A. (2018). Prevalence and characteristics of self-harm in adolescents: Meta-analyses of community-based studies 1990-2015. Journal of the American Academy of Child & Adolescent Psychiatry.

[bib0031] Glenn C.R., Kleiman E.M., Kearns J.C., Santee A.C., Esposito E.C., Conwell Y., Alpert-Gillis L.J. (2022). Feasibility and acceptability of ecological momentary assessment with high-risk suicidal adolescents following acute psychiatric care. Journal of Clinical Child & Adolescent Psychology.

[bib0032] Glenn C.R., Lanzillo E.C., Esposito E.C., Santee A.C., Nock M.K., Auerbach R.P. (2017). Examining the course of suicidal and nonsuicidal self-injurious thoughts and behaviors in outpatient and inpatient adolescents. Journal of Abnormal Child Psychology.

[bib0033] Grant A.M., Franklin J., Langford P. (2002). The Self-Reflection and Insight Scale: A new measure of private self-consciousness. Social Behavior and Personality: An International Journal.

[bib0034] Gromatsky M., Patel T.A., Wilson S.M., Mann A.J., Aho N., Carpenter V.L., Calhoun P.S., Beckham J.C., Goodman M., Kimbrel N.A. (2022). Qualitative analysis of participant experiences during an ecological momentary assessment study of nonsuicidal self-injury among veterans. Psychiatry Research.

[bib0035] Groschwitz R.C., Kaess M., Fischer G., Ameis N., Schulze U.M.E., Brunner R., Koelch M., Plener P.L. (2015). The association of non-suicidal self-injury and suicidal behavior according to DSM-5 in adolescent psychiatric inpatients. Psychiatry Research.

[bib0036] Haliczer L.A., Dixon-Gordon K.L. (2023). Social stressors, emotional responses, and NSSI urges and behaviors in daily life. Journal of Affective Disorders.

[bib0037] Hall M., Lappenbusch L.M., Wiegmann E., Rubel J.A. (2025). To use or not to use: Exploring therapists’ experiences with pre-treatment EMA-based personalized feedback in the TheraNet project. Administration and Policy in Mental Health and Mental Health Services Research.

[bib0038] Hasking P., Boyes M. (2018). The Non-Suicidal Self-Injury Expectancy Questionnaire: Factor structure and initial validation. Clinical Psychologist.

[bib0039] Hasking P., Rose A. (2016). A preliminary application of social cognitive theory to nonsuicidal self-injury. Journal of Youth and Adolescence.

[bib0040] Hasking P., Whitlock J., Voon D., Rose A. (2017). A cognitive-emotional model of NSSI: Using emotion regulation and cognitive processes to explain why people self-injure. Cognition and Emotion.

[bib0041] Hoelscher E.C., Victor S.E., Kiekens G., Ammerman B. (2025). Ethical considerations for the use of ecological momentary assessment in non-suicidal self-injury research. Ethics & Behavior.

[bib0042] Hughes C.D., King A.M., Kranzler A., Fehling K., Miller A., Lindqvist J., Selby E.A. (2019). Anxious and overwhelming affects and repetitive negative thinking as ecological predictors of self-injurious thoughts and behaviors. Cognitive Therapy and Research.

[bib0043] International Society for the Study of Self-Injury. (2024). *About self-injury*. https://www.itriples.org/aboutnssi.

[bib0044] Jacobucci R., Ammerman B.A., McClure K. (2024). Examining missingness at the momentary level in clinical research using ecological momentary assessment: Implications for suicide research. Journal of Clinical Psychology.

[bib0045] Janssens J.J., Kiekens G., Jaeken M., Kirtley O.J. (2024). A systematic review of interpersonal processes and their measurement within experience sampling studies of self-injurious thoughts and behaviours. Clinical Psychology Review.

[bib0046] Jennissen S., Huber J., Ehrenthal J.C., Schauenburg H., Dinger U. (2018). Association Between Insight and Outcome of Psychotherapy: Systematic Review and Meta-Analysis. American Journal of Psychiatry.

[bib0047] Jerusalem M., Schwarzer R. (1992). Self-efficacy: Thought control of action.

[bib0048] Kaess M., Edinger A., Fischer-Waldschmidt G., Parzer P., Brunner R., Resch F. (2020). Effectiveness of a brief psychotherapeutic intervention compared with treatment as usual for adolescent nonsuicidal self-injury: a single-centre, randomised controlled trial. European Child and Adolescent Psychiatry.

[bib0049] Kauer S.D., Reid S.C., Crooke A.H.D., Khor A., Hearps S.J.C., Jorm A.F., Sanci L., Patton G. (2012). Self-monitoring using mobile phones in the early stages of adolescent depression: randomized controlled trial. Journal of Medical Internet Research.

[bib0050] Kiekens G., Claes L., Kleiman E.M., Luyckx K., Coppersmith D.D.L., Fortgang R.G., Myin-Germeys I., Nock M.K. (2024). The short-term course of nonsuicidal self-injury among individuals seeking psychiatric treatment. JAMA Network Open.

[bib0051] Kiekens G., Claes L., Schoefs S., Kemme N.D.F.F., Luyckx K., Kleiman E.M., Nock M.K., Myin-Germeys I. (2023). The detection of acute risk of self-injury project: protocol for an ecological momentary assessment study among individuals seeking treatment. JMIR Research Protocols.

[bib0052] Kiekens G., Hasking P., Boyes M., Claes L., Mortier P., Auerbach R.P., Cuijpers P., Demyttenaere K., Green J.G., Kessler R.C., Myin-Germeys I., Nock M.K., Bruffaerts R. (2018). The associations between non-suicidal self-injury and first onset suicidal thoughts and behaviors. Journal of Affective Disorders.

[bib0053] Kiekens G., Hasking P., Bruffaerts R., Alonso J., Auerbach R.P., Bantjes J., Benjet C., Boyes M., Chiu W.T., Claes L., Cuijpers P., Ebert D.D., Mak A., Mortier P., O’Neill S., Sampson N.A., Stein D.J., Vilagut G., Nock M.K., Kessler R.C. (2023). Non-suicidal self-injury among first-year college students and its association with mental disorders: Results from the World Mental Health International College Student (WMH-ICS) initiative. Psychological Medicine.

[bib0054] Kiekens G., Hasking P., Claes L., Mortier P., Auerbach R.P., Boyes M., Cuijpers P., Demyttenaere K., Green J.G., Kessler R.C., Nock M.K., Bruffaerts R. (2018). The DSM-5 nonsuicidal self-injury disorder among incoming college students: Prevalence and associations with 12-month mental disorders and suicidal thoughts and behaviors. Depression and Anxiety.

[bib0055] Kiekens G., Hasking P., Nock M.K., Boyes M., Kirtley O., Bruffaerts R., Myin-Germeys I., Claes L. (2020). Fluctuations in Affective States and Self-Efficacy to Resist Non-Suicidal Self-Injury as Real-Time Predictors of Non-Suicidal Self-Injurious Thoughts and Behaviors. Frontiers in Psychiatry.

[bib0056] Kiekens G., Robinson K., Tatnell R., Kirtley O.J. (2021). Opening the black box of daily life in nonsuicidal self-injury research: With great opportunity comes great responsibility. JMIR Mental Health.

[bib0057] Kirtley O.J., Myin-Germeys I., Kuppens P. (2022). The Open Handbook of Experience Sampling Methodology. A step-by-step guide to designing, conducting, and analyzing ESM studies.

[bib0058] Kirtley, O. J., Eisele, G., Kunkels, Y., Hiekkaranta, A. P., Van Heck, L., Pihlajamäki, M., Kunc, B., Schoefs, S., Kemme, N. D. F., Biesemans, T., & Myin-Germeys, I. (2024). *The ESM item repository*. 10.17605/OSF.IO/KG376.

[bib0059] Kirtley O.J., Lafit G., Achterhof R., Hiekkaranta A.P., Myin-Germeys I. (2021). Making the black box transparent: a template and tutorial for registration of studies using experience-sampling methods. Advances in Methods and Practices in Psychological Science.

[bib100] Kirtley, O.J., Sohier, B., Simsa, B., Achterhof, R., Myin-Germeys, I., & Lafit, G.. (in press). Reactivity to experience sampling among adolescents with and without a lifetime or current history of self-harm thoughts or behaviours. *Psychological Assessment*. doi:10.31234/osf.io/tvw68. 10.1037/pas0001375 41196733

[bib0061] Kivelä L.M.M., Fiß F., van der Does W., Antypa N. (2024). Examination of acceptability, feasibility, and iatrogenic effects of ecological momentary assessment (EMA) of suicidal ideation. Assessment.

[bib0062] Kleiman E.M., Bentley K.H., Glenn C.R., Liu R.T., Rizvi S.L. (2022). Building on the past 50 years, not starting over: A balanced interpretation of meta-analyses, reviews, and commentaries on treatments for suicide and self-injury. General Hospital Psychiatry.

[bib0063] Kuburi S., Ewing L., Hamza C.A., Goldstein A.L. (2024). A daily diary study of the relation between stress and nonsuicidal self-injury and the moderating role of emotion dysregulation in emerging adulthood. Journal of Youth and Adolescence.

[bib0064] Kuehn K.S., Dora J., Harned M.S., Foster K.T., Song F., Smith M.R., King K.M. (2022). A meta-analysis on the affect regulation function of real-time self-injurious thoughts and behaviours. Nature Human Behaviour.

[bib0065] Law M.K., Furr R.M., Arnold E.M., Mneimne M., Jaquett C., Fleeson W. (2015). Does assessing suicidality frequently and repeatedly cause harm? A randomized control study. Psychological Assessment.

[bib0066] Linehan M.M. (2015).

[bib0067] Liu R.T. (2023). The epidemiology of non-suicidal self-injury: lifetime prevalence, sociodemographic and clinical correlates, and treatment use in a nationally representative sample of adults in England. Psychological Medicine.

[bib0068] Lovibond S.H, Lovibond P.F. (1995). Depression Anxiety Stress Scales (DASS-21, DASS-42). APA PsychTests.

[bib0069] McNeish D., Hamaker E.L. (2020). A primer on two-level dynamic structural equation models for intensive longitudinal data in Mplus. Psychological Methods.

[bib0070] Mestdagh M., Verdonck S., Piot M., Niemeijer K., Kilani G., Tuerlinckx F., Kuppens P., Dejonckheere E. (2023). m-Path: An easy-to-use and highly tailorable platform for ecological momentary assessment and intervention in behavioral research and clinical practice. Frontiers in Digital Health.

[bib0071] Millon E.M., Alqueza K.L., Kamath R.A., Marsh R., Pagliaccio D., Blumberg H.P., Stewart J.G., Auerbach R.P. (2022). Non-suicidal self-injurious thoughts and behaviors among adolescent inpatients. Child Psychiatry and Human Development.

[bib0072] Mink F., Lutz W., Hehlmann M.I. (2025). Ecological Momentary Assessment in psychotherapy research: A systematic review. Clinical Psychology Review.

[bib0073] Muthén B.O., Muthén L.K., Asparouhov T. (2017).

[bib0074] Myin-Germeys I., Kasanova Z., Vaessen T., Vachon H., Kirtley O., Viechtbauer W., Reininghaus U. (2018). Experience sampling methodology in mental health research: new insights and technical developments. World Psychiatry.

[bib0075] Myin-Germeys I., Schick A., Ganslandt T., Hajdúk M., Heretik A., Van Hoyweghen I., Kiekens G., Koppe G., Marelli L., Nagyova I., Weermeijer J., Wensing M., Wolters M., Beames J., de Allegri M., di Folco S., Durstewitz D., Katreniaková Z., Lievevrouw E., Reininghaus U. (2024). The experience sampling methodology as a digital clinical tool for more person-centered mental health care: an implementation research agenda. Psychological Medicine.

[bib0076] Nock M.K., Holmberg E.B., Photos V.I., Michel B.D. (2007). Self-injurious thoughts and behaviors interview: Development, reliability, and validity in an adolescent sample. Psychological Assessment.

[bib0077] Ose S.O., Tveit T., Mehlum L. (2021). Non-suicidal self-injury (NSSI) in adult psychiatric outpatients - A nationwide study. Journal of Psychiatric Research.

[bib0078] Pinheiro J., Bates D. (2023). nlme: Linear and Nonlinear Mixed Effects Models. R Package Version.

[bib0079] Plener P.L., Allroggen M., Kapusta N.D., Brähler E., Fegert J.M., Groschwitz R.C. (2016). The prevalence of Nonsuicidal Self-Injury (NSSI) in a representative sample of the German population. BMC Psychiatry.

[bib0080] Team Posit (2024).

[bib0081] Rintala A., Wampers M., Lafit G., Myin-Germeys I., Viechtbauer W. (2021). Perceived disturbance and predictors thereof in studies using the experience sampling method. Current Psychology.

[bib0082] Rintala A., Wampers M., Myin-Germeys I., Viechtbauer W. (2019). Response compliance and predictors thereof in studies using the experience sampling method. Psychological Assessment.

[bib0083] Rippon D., Shepherd J., Wakefield S., Lee A., Pollet T.V. (2024). The role of self-efficacy and self-esteem in mediating positive associations between functional social support and psychological wellbeing in people with a mental health diagnosis. Journal of Mental Health.

[bib0084] Robillard C.L., Claes L., Victor S.E., Myin-Germeys I., Kiekens G. (2025). Self-criticism is a real-time predictor of non-suicidal self-injury and disordered eating: An ecological momentary assessment study among treatment-seeking individuals. Journal of Affective Disorders.

[bib0085] Rockstroh F., Edinger A., Josi J., Brunner R., Resch F., Kaess M. (2023). Brief psychotherapeutic intervention compared with treatment as usual for adolescents with nonsuicidal self-injury: Outcomes over a 2-4-year follow-up. Psychotherapy and Psychosomatics.

[bib0086] Spangenberg L., Böhler L., Hoke T.-M., Serebriakova J., Eimen J., Forkmann T., Strauss M., Stengler K., Glaesmer H. (2025). Promising or discouraging? Potentials and reactivity of real-time data collection in monitoring suicide-related thoughts and behaviors over weeks and months. Participants' views on ecological momentary assessments and wearable use. Digital Health.

[bib0087] Spitzen T.L., Tull M.T., Gratz K.L. (2022). The roles of emotion regulation self-efficacy and emotional avoidance in self-injurious thoughts and behaviors. Archives of Suicide Research.

[bib0088] Stone A.A., Shiffman S. (1994). Ecological Momentary Assessment (Ema) in behavioral medicine. Annals of Behavioral Medicine.

[bib0089] Swannell S.V., Martin G.E., Page A., Hasking P., St John N.J. (2014). Prevalence of nonsuicidal self-injury in nonclinical samples: Systematic review, meta-analysis and meta-regression. Suicide and Life-Threatening Behavior.

[bib0090] Tatnell R., Kelada L., Hasking P., Martin G. (2014). Longitudinal analysis of adolescent NSSI: The role of intrapersonal and interpersonal factors. Journal of Abnormal Child Psychology.

[bib0091] Teeuw, B., & Schwarzer, R. (1994). *Dutch Adaptation of the General Self-Efficacy Scale*. 1994.

[bib0092] Turner B.J., Cobb R.J., Gratz K.L., Chapman A.L. (2016). The role of interpersonal conflict and perceived social support in nonsuicidal self-injury in daily life. Journal of Abnormal Psychology.

[bib0093] Vachon H., Viechtbauer W., Rintala A., Myin-Germeys I. (2019). Compliance and retention with the experience sampling method over the continuum of severe mental disorders: Meta-analysis and recommendations. Journal of Medical Internet Research.

[bib0094] van Alphen N.R., Stewart J.G., Esposito E.C., Pridgen B., Gold J., Auerbach R.P. (2017). Predictors of rehospitalization for depressed adolescents admitted to acute psychiatric treatment. The Journal of Clinical Psychiatry.

[bib0095] van Roekel E., Keijsers L., Chung J.M. (2019). A review of current ambulatory assessment studies in adolescent samples and practical recommendations. Journal of Research on Adolescence.

[bib0096] Weermeijer J., Kiekens G., Wampers M., Kuppens P., Myin-Germeys I. (2024). Practitioner perspectives on the use of the experience sampling software in counseling and clinical psychology. Behaviour & Information Technology.

[bib0097] Weermeijer J.D.M., Wampers M., de Thurah L., Bonnier R., Piot M., Kuppens P., Myin-Germeys I., Kiekens G. (2023). Usability of the experience sampling method in specialized mental health care: Pilot evaluation study. JMIR Formative Research.

[bib0098] Wester K., Trepal H., King K. (2018). Nonsuicidal self-injury: Increased prevalence in engagement. Suicide and Life-Threatening Behavior.

[bib0099] Wilkinson P.O., Qiu T., Neufeld S., Jones P.B., Goodyer I.M. (2018). Sporadic and recurrent non-suicidal self-injury before age 14 and incident onset of psychiatric disorders by 17 years: Prospective cohort study. The British Journal of Psychiatry : The Journal of Mental Science.

