# Supplementary material for: Clinical benefits and challenges of ecological momentary assessment in individuals who self-injure and seek mental health treatment
Source: Int J Clin Health Psychol. 2025 Sep 23;25(3):100618. doi: 10.1016/j.ijchp.2025.100618 (PMC12492036; doi:10.1016/j.ijchp.2025.100618)
Supplement: Supplementary file 1 [file mmc1.docx]

# Supplementary Materials:

**Supplementary Table 1**.

EMA items included in the daily questionnaires.

| **EMA Items** | | | |
| --- | --- | --- | --- |
| **Variable** | **Dutch Item** | **English Translation** | **Answer Options** |
| Beep Disturbance | ‘Ik vond deze beep storend.’ | ‘I found this beep annoying.’ | Seven point scale (0= not at all, to 6= very much) |
| NSSI^a.^ thoughts (retrospectively) | Sinds de laatste beep, dacht je eraan jezelf opzettelijk te verwonden zonder de wens om je leven te beëindigen? | ‘Since the last beep, did you think about intentionally injuring yourself without wanting to end your life? | Seven point scale (0= not at all, to 6= very much) |
| NSSI urges | ‘Op dit moment, hoe sterk is de drang aanwezig om jezelf te verwonden zonder de wens om je leven te beëindigen?’ | ‘In this moment, how strong is the urge to injure yourself without wanting to end your life?’ | Seven point scale (0= absent, to 6= very strong) |
| Self-Efficacy to resist NSSI | ‘ Op dit moment, hoe zeker ben je ervan dat je kan weerstaan om jezelf opzettelijk te verwonden? | ‘In this moment, how certain are you in your ability to resist injuring yourself?’ | Seven point scale (0= not at all, to 6= very) |
| NSSI behaviors (retrospectively) | ‘Sinds de laatste beep, heb je jezelf opzettelijk verwond zonder de wens om je leven te beëindigen? (bijvoorbeeld jezelf gesneden, gekrabd, of geslagen.’ | ‘Since the last beep, did you intentionally injured yourself without wanting to end your life? (for example cutting, scratching or hitting yourself.’ | No (=0)/Yes(=1) |

Note: ^a^ Non-Suicidal Self-Injury (NSSI).

**Supplementary Table** **2**.

Score distribution of the general self-insight items with the modes indicated.

| **General Self-Insight (n=98)** | | | | | | |
| --- | --- | --- | --- | --- | --- | --- |
| **Item** | **Score**  **N (%)** | | | | | |
|  | **1 = Totally disagree** | **2 = Disagree** | **3= Somewhat disagree** | **4= Somewhat agree** | **5= Agree** | **6 = Totally agree** |
| The self-monitoring made me more aware of my feelings and thoughts. | 0 (0%) | 0 (0%) | 5 (5.10%) | 20 (20.41%) | 52(53.06%) | 21 (21.43%) |
| *Completing the app often confused me about how I feel about things. | 2 (2.04%) | 19 (19.39%) | 22 (22.45%) | 30 (30.61%) | 20 (20.41%) | 5 (5.10%) |
| The self-monitoring made it clear why I behave in certain ways. | 2 (2.04%) | 17 (17.35%) | 31 (31.63%) | 32 (32.65%) | 12 (12.24%) | 4 (4.08%) |
| The self-monitoring helped me to understand my feelings and thoughts better. | 2 (2.04%) | 14 (14.29%) | 21 (21.43%) | 40 (40.82%) | 17 (17.35%) | 4 (4.08%) |
| The self-monitoring helped me to understand my behavior better. | 2 (2.04%) | 18 (18.37%) | 16 (16.33%) | 42 (42.86%) | 17 (17.35%) | 3 (3.06%) |
| *Thinking about my thoughts while completing the questions confused me. | 3 (3.06%) | 20 (20.41%) | 18 (18.37%) | 35 (35.71%) | 19 (19.39%) | 3 (3.06%) |
| The self-monitoring helped me to better understand who I am. | 10 (10.20%) | 28 (28.57%) | 27 (27.55%) | 21 (21.43%) | 10 (10.20%) | 2 (2.04%) |
| With the help of the app I better understood why I feel the way I do about people around me. | 5 (5.10%) | 19 (20.39%) | 20 (20.41%) | 42 (43.86%) | 9 (9.18%) | 3 (3.06%) |

*Note*: * Reverse-scored items in the calculation of the mean score

**Supplementary Table 3.**

Score distribution of the NSSI^a.^-specific self-insight items with the modes indicated.

| **NSSI-specific self-insight (n=96)** | | | | | | | | | | |
| --- | --- | --- | --- | --- | --- | --- | --- | --- | --- | --- |
| **Item** | **Score**  **N (%)** | | | | | | | | | |
|  | **1 = Strongly disagree** | **2** | **3** | **4** | **5** | **6** | **7** | **8** | **9** | **10 = Strongly agree** |
| The self-monitoring made me more aware of my triggers for self-injury | 3 (3.13%) | 0 (0%) | 3 (3.13%) | 6 (6.25%) | 16 (16.67%) | 18 (18.75%) | 30 (31.25%) | 19 (19.79%) | 0 (0%) | 1 (1.04%) |
| The self-monitoring helped me to better understand why I think about injuring myself | 5 (5.21%) | 5 (5.21%) | 3 (3.13%) | 4 (4.17%) | 10 (10.42%) | 23 (23.96%) | 25 (26.04%) | 15 (15.63%) | 5 (5.21%) | 1 (1.04%) |
| The self-monitoring helped me to become more aware of why I feel an urge to self-injure in in certain situations. | 3 (3.13%) | 3 (3.13%) | 4 (4.17%) | 1 (1.04%) | 11 (11.46%) | 22 (22.92%) | 25 (26.04%) | 21 (21.88%) | 5 (5.21%) | 1 (1.04%) |
| The self-monitoring helped me to better understand why I sometimes do and sometimes do not self-injure myself in high risk situations. | 2 (2.08%) | 0 (0%) | 10 (10.42%) | 7 (7.29%) | 16 (16.67%) | 19 (19.79%) | 20 (20.83%) | 16 (16.67%) | 4 (4.17%) | 2 (2.08%) |
| The self-monitoring helped me to become more aware of triggers for other self-damaging thoughts and behaviors (e.g., (binge eating, purging, binge drinking, suicidal thoughts). | 3 (3.13%) | 3 (3.13%) | 7 (7.29%) | 4 (4.17%) | 13 (13.54%) | 20 (20.83%) | 28(29.17%) | 8 (8.33%) | 6 (6.25%) | 4 (4.17%) |

Note: ^a^ Non-Suicidal Self-Injury (NSSI)

**Supplementary Table 4**.

Score distribution of the general self-efficacy items with the modes indicated.

| **General Self-Efficacy (n=97)** | | | | |
| --- | --- | --- | --- | --- |
| **Item** | **Score**  **N (%)** | | | |
|  | **1 = Not at all true** | **2 = Barely true** | **3= Moderately true** | **4= Exactly true** |
| By what I learned about myself through the self-monitoring, I feel that I am better able to solve difficult problems if I try hard enough. | 11 (11.34%) | 40 (41.24%) | 42 (43.30%) | 4 (4.12%) |
| By what I learned about myself during through the self-monitoring, I am better able to achieve I want to achieve despite someone working against me. | 11 (11.34%) | 56 (57.73%) | 29 (29.90%) | 1 (1.03%) |
| The self-monitoring made it easier for me to stick to my plans and achieve my goals. | 12 (12.37%) | 47 (48.45%) | 34 (35.05%) | 4 (4.12%) |
| The self-monitoring helped me to deal with unexpected events more effectively. | 13 (13.40%) | 46 (47.42%) | 35 (36.08%) | 3 (3.09%) |
| By what I learned about myself through the self-monitoring, , I know better how to act in unforeseen situations. | 16 (16.49%) | 42 (43.30%) | 35 (36.08%) | 4 (4.12%) |
| By what I learned about myself through the self-monitoring, I feel that I am better able to solve difficult problems if I try hard enough | 11 (11.34%) | 43 (44.33%) | 38 (39.18%) | 5 (5.15%) |
| The self-monitoring helped me to keep more calm when faced with difficulties because I trust my ability to solve problems more. | 17 (17.52%) | 43 (44.33%) | 33 (34.02%) | 4 (4.12%) |
| By what I learned about myself through the self-monitoring, I now have multiple solutions when faced with a problem. | 15 (15.46%) | 53 (54.64%) | 28 (28.87%) | 1 (1.03%) |
| By what I learned about myself through the self-monitoring, I now know better what to do when I am in a predicament. | 15 (15.46%) | 50 (51.55%) | 28 (28.87%) | 4 (4.12%) |
| Whatever happens, what I have learned about myself through self-monitoring has given me more confidence I will get through it. | 14 (14.43%) | 33 (34.02%) | 44 (45.36%) | 6 (6.19%) |

**Supplementary Table 5.**

Score distribution of the NSSI^a^-specific self-efficacy items with the modes indicated.

| **NSSI^a.^-specific self-efficacy (n=96)** | | | | | | | | | | |
| --- | --- | --- | --- | --- | --- | --- | --- | --- | --- | --- |
| **Item** | **Score**  **N (%)** | | | | | | | | | |
|  | **1 = Strongly disagree** | **2** | **3** | **4** | **5** | **6** | **7** | **8** | **9** | **10 = Strongly agree** |
| The self-monitoring helped me to improve my confidence in my ability to resist the urge to injure myself. | 5 (5.21%) | 5 (5.21%) | 4 (4.17%) | 11 (11.46%) | 9 (9.38%) | 23 (23.96%) | 16 (16.67%) | 14 (14.58%) | 6 (6.25%) | 3 (3.13%) |
| The self-monitoring helped me to feel I am now better at avoiding high-risk situations in a timely manner. | 5 (5.21%) | 5 (5.21%) | 12 (12.50%) | 14 (14.58%) | 23 (23.96%) | 16 (16.67%) | 10 (10.42%) | 8 (8.33%) | 2 (2.08%) | 1 (1.04%) |
| The self-monitoring helped me to know better what to do when I feel an urge to self-injure | 7 (7.29%) | 5 (5.21%) | 10 (10.42%) | 11 (11.46%) | 20 (20.83%) | 20 (20.83%) | 11 (11.46%) | 10 (10.42%) | 1 (1.04%) | 1 (1.04%) |
| The self-monitoring helped me to feel more in control of my self-injury. | 5 (5.21%) | 4 (4.17%) | 9 (9.38%) | 6 (6.25%) | 13 (13.54%) | 19 (19.79%) | 15 (15.62%) | 11 (11.46%) | 8 (8.33%) | 6 (6.25%) |
| The self-monitoring helped me to become more confident in my ability to resist other self-damaging thoughts and behaviors (e.g., (binge eating, purging, binge drinking, and suicidal thoughts). | 6 (6.25%) | 8 (8.33%) | 5 (5.21%) | 10 (10.42%) | 23 (23.96%) | 13 (13.54%) | 8 (8.33%) | 12 (12.50%) | 4 (4.17%) | 7 (7.29%) |

Note: ^a^ Non-Suicidal Self-Injury (NSSI)

**Supplementary Table 6.**

Score distribution of the Emotional Discomfort items with the modes indicated.

| **Emotional Discomfort (n=96)** | | | | | |
| --- | --- | --- | --- | --- | --- |
| **item** | **Score**  **N (%)** | | | | |
|  | **1 = totally disagree** | **2 = disagree** | **3 = neutral** | **4 = Agree** | **5 = Totally agree** |
| I got emotionally upset at times filling out the questions in the smartphone study. | 8 (8.33%) | 13 (13.54%) | 14 (14.58%) | 42 (43.75%) | 19 (19.79%) |
| Answering the self-monitoring questions in the smartphone study caused me stress. | 2 (2.08%) | 11 (11.46%) | 31 (32.29%) | 43 (44.79%) | 9 (9.38%) |
| *I enjoyed participating in the smartphone study.^a.^ | 1 (1.04%) | 6 (6.25%) | 33 (34.38%) | 42 (43.75%) | 14 (14.58%) |
| Participating in the smartphone study was tiring | 8 (8.33%) | 8 (8.33%) | 26 (27.08%) | 34 (35.42%) | 20 (20.83%) |

Note: ^a^* Reverse-scored in the calculation of the mean score.

**Supplementary Table 7.**

*Unstandardized contemporaneous associations of NSSI cognitions and emotion dysregulation predicting beep disturbance within the same assessment (n=98, 12,327 surveys).*

|  | **Contemporaneous Model 1:**  Emotion dysregulation and beep disturbance | **Contemporaneous Model 2:**  NSSI thoughts and beep disturbance | **Contemporaneous Model 3:**  NSSI urges and beep disturbance | **Contemporaneous**  **Model 4:**  Self-efficacy to resist NSSI and beep disturbance |
| --- | --- | --- | --- | --- |
| **Average effect in the population** | *B* (95% CrI) | *B* (95% CrI) | *B* (95% CrI) | *B* (95% CrI) |
| Beep disturbance | **1.609 (1.358; 1.859)** | **1.608 (1.341; 1.871)** | **1.612 (1.358; 1.859)** | **1.611 (1.359; 1.857)** |
| State variable | **2.168 (1.938; 2.398)** | **1.651 (1.401; 1.887)** | **1.592 (1.349; 1.835)** | **4.422 (4.196; 4.645)** |
| Slope of state variable predicting beep disturbance with the same assessment | **0.053 (0.029; 0.078)** | 0.015 (-0.016; 0.046) | 0.021 (-0.018; 0.058) | -0.025 (-0.062; 0.012) |

*Note:.* Each column represents the results of a bivariate multilevel vector autoregressive model in the residual dynamic structural equation framework with the autoregressive parameters and person-specific residual variance of momentary variables not shown here. Boldface indicates a 95% probability that the true value of the impact is not null (i.e., the credibility interval does not include zero). *B* = Median Point Estimate, 95% CrI = 95% Credibility Interval, Posterior SD = Posterior Standard Deviation; NSSI = Non-Suicidal Self-Injury. Full model output can be consulted on the OSF project page.

**Supplementary Table 8.**

*Unstandardized temporal associations between emotion dysregulation and beep disturbance across two hours between assessments (n=98, 12,327 assessments).*

|  | **Temporal Model 1:**  Emotion dysregulation and beep disturbance |
| --- | --- |
|  | Bivariate model |
| **Average effects in the population** | *B* (95% CrI) |
| Beep disturbance | **1.608 (1.354; 1.881)** |
| Emotion dysregulation | **2.163 (1.942; 2.400)** |
| Autoregression beep disturbance_t-2hours_ | **0.284 (0.237; 0.330)** |
| Autoregression emotion of dysregulation_t-2hours_ | **0.400 (0.358; 0.440)** |
| Slope beep of disturbance_t-2hours_ predicting emotion dysregulation | **0.035 (0.006; 0.063)** |
| Slope of emotion dysregulation_t-2hours_ predicting disturbance | **0.040 (0.018; 0.062)** |

*Note:* Represents the results of a multilevel vector autoregressive model in the dynamic structural equation framework with the person-specific residual variance of emotion dysregulation and beep disturbance not shown here. Boldface indicates a 95% probability that the true value of the impact is not null (i.e., the credibility interval does not include zero). *B* = Median Point Estimate, 95% CrI = 95% Credibility Interval, Posterior SD = Posterior Standard Deviation; NSSI = Non-Suicidal Self-Injury. Full model output can be consulted on the OSF project page.

**Supplementary Table 9.**

*Unstandardized temporal associations between NSSI cognitions and beep disturbance across two hours between assessments (n=98, 12,327 assessments).*

|  | **Temporal Models 2:**  NSSI thoughts and beep disturbance | | **Temporal Models 3:**  NSSI urges and beep disturbance | | **Temporal Models 4:**  Self-efficacy to resist NSSI and beep disturbance | |
| --- | --- | --- | --- | --- | --- | --- |
|  | Bivariate model | Multivariate model (controlling for emotion dysregulation) | Bivariate model | Multivariate model (controlling for emotion dysregulation) | Bivariate model | Multivariate model (controlling for emotion dysregulation) |
| **Average effects in the population** | *B* (95% CrI) | *B* (95% CrI) | *B* (95% CrI) | *B* (95% CrI) | *B* (95% CrI) | *B* (95% CrI) |
| Beep disturbance | **1.619 (1.358; 1.881)** | **1.615 (1.368; 1.877)** | **1.611 (1.358; 1.885)** | **1.608 (1.363; 1.869)** | **1.610 (1.356; 1.881)** | **1.607 (1.359; 1.868)** |
| NSSI cognition | **1.655 (1.413; 1.891)** | **1.649 (1.411; 1.908)** | **1.589 (1.356; 1.842)** | **1.591 (1.345; 1.825)** | **4.423 (4.203; 4.651)** | **4.426 (4.197; 4.643)** |
| Emotion dysregulation | **-** | **2.177 (1.953; 2.394)** | **-** | **2.160 (1.933; 2.372)** | **-** | **-** |
| Autoregression beep disturbance_t-2hours_ | **0.282 (0.234; 0.329)** | **0.273 (0.226; 0.320)** | **0.279 (0.233; 0.324)** | **0.269 (0.224; 0.316)** | **0.282 (0.237; 0.328)** | **0.274 (0.228; 0.321)** |
| Autoregression NSSI cognition_t-2hours_ | **0.415 (0.363; 0.466)** | **0.343 (0.294; 0.391)** | **0.398 (0.350; 0.442)** | **0.329 (0.284; 0.372)** | **0.403 (0.359; 0.448)** | **0.344 (0.300; 0.387)** |
| Autoregression emotion dysregulation_t-2hours_ | **-** | **0.420 (0.375; 0.462)** | **-** | **0.421 (0.377; 0.466)** | **-** | **0.414 (0.370; 0.460)** |
| Slope of beep disturbance_t-2hours_  predicting NSSI cognition | **0.044 (0.015; 0.074)** | **0.035 (0.005; 0.064)** | 0.028 (-0.007; 0.061) | 0.019 (-0.015; 0.053) | -0.017 (-0.038; 0.003) | -0.011 (-0.031; 0.008) |
| Slope of NSSI cognition_t-2hours_  predicting beep disturbance | **0.031 (0.007; 0.055)** | 0.019 (-0.004; 0.041) | **0.047 (0.020; 0.076)** | **0.031 (0.003; 0.061)** | **-0.044 (-0.073; -0.017)** | **-0.027 (-0.055; -0.001)** |
| Slope of emotion dysregulation_t-2hours_  predicting NSSI cognition | **-** | **0.143 (0.115; 0.171)** | **-** | **0.126 (0.097; 0.157)** | **-** | **-0.092 (-0.122; -0.062)** |
| Slope of emotion dysregulation_t-2hours_  predicting beep disturbance | **-** | **0.032 (0.009; 0.056)** | **-** | **0.031 (0.009; 0.056)** | **-** | **0.030 (0.009; 0.055)** |

*Note:.* Each column represents the results of a multilevel vector autoregressive model in the dynamic structural equation framework with the person-specific residual variance of NSSI cognitions and beep disturbance not shown here. Boldface for average effects indicates a 95% probability that the true value of the impact is not null (i.e., the credibility interval does not include zero). *B* = Median Point Estimate, 95% CrI = 95% Credibility Interval, Posterior SD = Posterior Standard Deviation; NSSI = Non-Suicidal Self-Injury. Full model output can be consulted on the OSF project page.

**Supplementary Table 10.**

*Unstandardized temporal associations between NSSI behavior and beep disturbance across two hours between assessments (n=98, 12,327 assessments).*

|  | **Temporal Model 5:**  NSSI behavior and beep disturbance |
| --- | --- |
|  | Bivariate model |
| **Average effects in the population** | *B* (95% CrI) |
| Beep disturbance | **1.570 (1.308; 1.834)** |
| NSSI behavior (threshold) | **2.022 (1.847; 2.217)** |
| Autoregression of beep disturbance_t-2hours_ | **0.244 (0.197; 0.291)** |
| Autoregression of NSSI behavior_t-2hours_ | **0.347 (0.225; 0.465)** |
| Slope of beep disturbance_t-2hours_  on NSSI behavior | 0.025 (-0.028; 0.071) |
| Slope of NSSI behavior_t-2hours_  on beep disturbance | -0.013 (-0.062; 0.034) |

*Note:* Represents the results of a multilevel vector autoregressive model in the dynamic structural equation framework with the person-specific residual variance of beep disturbance not shown here. Boldface for average effects indicates a 95% probability that the true value of the impact is not null (i.e., the credibility interval does not include zero). *B* = Median Point Estimate, 95% CrI = 95% Credibility Interval, Posterior SD = Posterior Standard Deviation; NSSI = Non-Suicidal Self-Injury. Full model output can be consulted on the OSF project page.
